# Depressive symptom networks in the UK general adolescent population and in those looked after by local authorities

**DOI:** 10.1136/bmjment-2023-300707

**Published:** 2023-09-01

**Authors:** Pascal Schlechter, Tamsin Ford, Sharon A S Neufeld

**Affiliations:** Psychiatry, University of Cambridge, Cambridge, UK

**Keywords:** depression & mood disorders

## Abstract

**Background:**

Despite the importance of understanding depressive symptom constellations during adolescence and specifically in looked-after children, studies often only apply sum score models to understand depression in these populations, neglecting associations among single symptoms that can be elucidated in network analysis. The few network analyses in adolescents have relied on different measures to assess depressive symptoms, contributing to inconsistent cross-study results.

**Objective:**

In three population-based studies using the Short Mood and Feelings Questionnaire, we used network analyses to study depressive symptoms during adolescence and specifically in looked-after children.

**Method:**

We computed cross-sectional networks (Gaussian Graphical Model) in three separate datasets: the Mental Health of Children and Young People in Great Britain 1999 survey (n=4235, age 10–15 years), the mental health of young people looked after by local authorities in Great Britain 2002 survey (n=643, age 11–17 years) and the Millennium Cohort Study in the UK 2015 (n=11 176, age 14 years).

**Findings:**

In all three networks, *self-hate* emerged as a key symptom, which aligns with former network studies. *I was no good anymore* was also among the most central symptoms. Among looked-after children, *I was a bad person* constituted a central symptom, while this was among the least central symptom in the other two datasets. The Diagnostic and Statistical Manual of Mental Disorders, Fifth Edition symptom *I did not enjoy anything* was not central.

**Conclusions:**

Findings indicate that looked-after children’s depressive symptoms may be more affected by negative self-evaluation compared with the general population.

**Clinical implications:**

Intervention efforts may benefit from being tailored to negative self-evaluations.

**WHAT IS ALREADY KNOWN ON THIS TOPIC**

- Adolescence is a critical developmental period marked by significant psychobiological transitions and life events that influence the presentation of depressive symptoms.
- The unique life circumstances of looked-after children render them especially vulnerable to experiencing depressive symptoms.
- While prior network analyses have indicated that depressive symptom constellations may differ in adolescents compared with adults, these findings have exhibited substantial inconsistencies and have not investigated looked-after children specifically.

**WHAT THIS STUDY ADDS**

- Using two population-based samples in the UK, and a sample focused on looked-after children, this study clarifies that adolescents’ depressive symptom networks are centrally shaped by negative self-evaluations, and that looked-after children suffer from more impaired self-worth than their peers.

**HOW THIS STUDY MIGHT AFFECT RESEARCH, PRACTICE OR POLICY**

- Our findings highlight the pressing need to prioritise core symptoms related to negative self-evaluations when designing and testing interventions intended to mitigate depressive symptoms in adolescents.

## Background

In most countries around the world, depressive symptom incidence increases from childhood onwards, with new onset cases peaking around age 21 years.^1^ Meta-analyses estimate a worldwide prevalence of any depressive disorder of 2.6% (95% CI 1.7 to 3.9) for children and adolescents.^2^ Adolescence is characterised by profound psychobiological changes and major life events.^3^ Therefore, we need to understand the development of depressive symptoms during this sensitive developmental phase.^1^ Depressive symptoms may present differently as a function of age^4^ or life circumstances. Specifically, looked-after children, defined as young people in the care of the local authority, had an OR of 2.28 for having depression compared with those living in a private household.^5^ Looked-after children experience poorer mental health and educational attainment, more learning disabilities and special educational needs and suffer more adverse experiences, including exclusion from mainstream schools or stigmatisation by other pupils.^5–7^ This population is further at risk of childhood maltreatment, which is associated with both higher risk of depression^8^ and unfavourable treatment outcomes.^9^ Accordingly, for mental health services to be effective, depressive symptom presentations need to be understood accurately during development and specifically for looked-after children.

Potential developmental differences in the presentation of depression are not currently reflected in nosological classifications of the Diagnostic and Statistical Manual of Mental Disorders, Fifth Edition (DSM-5) and International Classification of Diseases (ICD)-11, as neither vary substantially in their disorder specification as a function of development.^10^ Only the DSM describes *irritability* rather than *depressed mood* as an alternate hallmark symptom for children and adolescents. Sum score models that assume symptoms are interchangeable indicators of an underlying common cause are thus often applied to measure depression during adolescence and specifically in looked-after children.^1 5^

To understand the presentation of adolescent depressive symptoms better, network analytic methods have been applied.^11^ The network perspective conceptualises a disorder as a causal system emerging from complex interactions among symptoms.^12^ The importance of symptoms is indicated by their centrality or interconnectedness, and core symptoms can reflect clinically relevant targets.^12 13^

However, few studies have examined properties of depression networks during adolescence.^14^ Analyses in adult samples indicated that *sadness* and *loss of interest* were central depressive symptoms, consistent with nosological systems.^11^ The few studies in children and adolescents point to different symptom profiles in these ages compared with adulthood,^15^ which has important implications for the nosological understanding of depression. Table 1 summarises the main findings from previous studies, with a broad range of core symptoms emerging across different populations using different questionnaires: *self-hatred, loneliness, sadness, pessimism, fatigue, self-deprecation, crying, school dislike, low self-esteem, low mood*, *feelings of worthlessness* and *no good anymore*. This research sheds light on symptom centrality and connectivity across different developmental stages, identifying core symptoms often not covered by the DSM or ICD.^15^ The identification of heterogenous core symptoms in children and adolescents aligns with the overall wide range of depressive symptoms observed clinically in this age range.^1^ Core symptoms during adolescence appear to encompass negative self-evaluation and may reflect endorsement of the DSM-5 depressive symptom covering feelings of worthlessness.^10^

**Table 1:** Overview of depression network studies in children and adolescents

Study	Sample/Measure	Main finding
Mullarkey *et al*^14^	High schools in urban and suburban areas in the USA N=1409 adolescents aged 13–19 years Children’s Depression Inventory (CDI)	Most central symptoms: *self-hatred, loneliness, sadness* and *pessimism*
Gijzen *et al*^19^	Community sample in the USA N=5888 adolescents aged 11–16 years CDI	Most central symptoms: *loneliness, sadness, self-hatred, fatigue, self-deprecation* and *crying*
Kim *et al*^20^	South-Korean community sample N=10 233 elementary school children aged 6–12 years CDI	Most central symptoms: *loneliness, self-hatred, school dislike* and *low self-esteem*
Manfro *et al*^15^	Two school-based samples from Brazil, aged 14–16 years N=7720 completed the Patient Health Questionnaire-9 (PHQ-9) N=1070 completed the Mood and Feelings Questionnaire (MFQ)	Most central symptoms PHQ-9: *low mood* and *feelings of worthlessness* Most central symptoms MFQ: *self-hatred* and *loneliness*
Mullarke *et al*^17^	Community sample in the USA N=1059 adolescents aged 15 years Short MFQ (SMFQ)	Most central symptoms: *self-hatred, loneliness, sadness* and *no good anymore*
Xie *et al*^18^	Chinese adolescents, mean ages of 14–16 years Three samples completed the PHQ-9 (n=1610), SMFQ (n=2194) and CDI (n= 571), respectively	Most central symptoms: *sadness, no good anymore* and *self-hatred*
Gossage *et al*^21^	New Zealand born, Pacific adolescents N=561; mean age 17 years CDI	Most central symptoms: loneliness, self-hatred and sadness

Previous network studies have used different measures of depression, limiting their comparability. A consistent instrument must be used to elucidate possible differences in network structures across different populations. The self-reported Moods and Feelings Questionnaire (MFQ) is recommended by the National Institute for Health and Clinical Excellence guidelines as a screening tool for childhood and adolescent depression.^16^ As shown in table 1, only three studies have used the MFQ or the short MFQ (SMFQ) to conduct network analysis.^15 17 18^ In these studies, *self-hate, sadness, loneliness* and *I was no good anymore* constituted the most central symptoms. The former three items also emerged as central in an additional three studies using the Children’s Depression Inventory,^17–21^ indicating their potential importance for intervention targets to remedy further symptom escalation.^13^ These symptoms may indicate cognitions and perceptions that constitute the development of self-worth.^10^ In addition, adolescence is a period of increased vulnerability to loneliness with detrimental mental health consequences.^22^ Accordingly, the interplay of these symptoms may be key for understanding depressive symptoms in adolescence.

However, symptom presentations may differ for looked-after children, who are in the care of local authorities for a variety of reasons including physical, sexual or emotional abuse or neglect and other circumstances that hinder parents’ caregiving, such as incarceration.^23^ These experiences are likely to lead to a dysfunctional attachment style in the young person, which can in turn lead to repeated experiences of negative interactions and unmet emotional needs throughout the young person’s life.^24^ While stable care can restore secure attachment to a certain degree,^25^ looked-after children show lower levels of secure attachment and higher levels of insecure and disorganised attachment compared with the general population.^24 26^ Indeed, looked-after children are at great risk of negative financial, educational, legal and health outcomes.^5 7^ Identification of core symptoms is paramount to better understand the symptom presentation in this vulnerable population. It could be that these life circumstances result in elevated symptoms, but that underlying symptom presentation in looked-after children otherwise remains equivalent to the general population. This would indicate that similar approaches for symptom reduction could be used in this vulnerable population as in the general population. Alternatively, different depressive symptoms may play a greater role in the network structure of looked-after children compared with the general population. For instance, *concentration problems* or seeing oneself as a *bad person* may constitute more central symptoms in looked-after children,^6^ as attention deficit hyperactivity disorder and conduct disorders are also more prevalent in this population.^5^

## Objectives

Our aims were twofold. First, we aimed to understand depressive symptom constellations in adolescents across two population-based UK studies. Second, we sought to understand whether depressive symptom networks differed in looked-after children compared with these general population samples. Using data from the Mental Health of Children and Young People in Great Britain (MHCYP), the Millennium Cohort Study (MCS) and the mental health of young people looked after by local authorities in Great Britain (LAC^5 23^), we investigated depressive symptoms measured with the SMFQ in three different studies, representing different UK populations regarding experienced adversities. Separate analyses per sample aimed to elucidate network structure replicability across populations with different demographic backgrounds. As former studies showed central symptoms of *self-hate, sadness, I was no good anymore* and *loneliness*,^15 17 18^ we expected these symptoms to be central in our analyses. We investigated exploratorily whether the symptom structure in the LAC sample differed from the other two samples.

## Methods

### Datasets

The 1999 MHCYP survey is representative of children from the general population of England, Scotland and Wales.^23^ A total of 4235 children aged 10–15 years responded to the SMFQ (mean age=12.95 years; table 2).

**Table 2:** Demographic characteristics

	MHCYP (n=4235)	MCS (n=11 176)	LAC (n=643)
Age			
Mean	12.95 (SD=1.41)	13.77 (SD=0.45)	11.30 years (SD=3.4),
Range	10–15	13–15	11–17 (n=473 between ages 11 and 15 years)
Gender*			
Male	2132 (50.3%)	5534 (49.5%)	359 (55.8%)
Female	2098 (49.5%)	5642 (50.5%)	280 (43.5%)
Ethnicity*			
White	3858 (91.1%)	8979 (80.3%)	583 (90.6%)
Non-white	371 (8.8%)	2113 (18.9%)	56 (8.7%)
Type of placement			
With foster parent(s)	–	–	397 (61.2%)
With natural parent(s)	–	–	79 (12.3%)
Other relative(s)	–	–	26 (4.0%)
Friend(s)	–	–	1 (0.01%)
Community home/other residential	–	–	119 (18.4%)
Living independently	–	–	17 (2.6%)

MHCYP participants were younger than MCS, p<0.001; LAC participants were younger than participants from both other cohorts, compared with both other samples, looked-after children were more likely male, p<0.001 and MCS participants were more likely non-white, p<0.001. All other comparisons were non-significant (p>0.32).

*Numbers which do not add up to 100% contain missing information.

LAC, looked after by local authorities; MCS, Millennium Cohort Study; MHCYP, Mental Health of Children and Young People in Great Britain.

The MCS is a longitudinal cohort study following young people born in the UK in 2000–2001.^27^ Children from ethnic minority backgrounds and families living in disadvantaged circumstances were oversampled. We took data from wave 6 when participants were aged 14 years (2015, n=11 176), when SMFQ was collected closest in age to MHCYP data. Predictors of attrition over time included disadvantaged families (eg, lower socioeconomic status, greater neighbourhood deprivation), ethnic minorities and single parent households.^28^

In LAC, data from three nationally representative surveys from 2002 to 2003 were combined (one each in England, Scotland and Wales) to gather data on looked-after children.^5 23^ Random samples of looked-after children were selected from the relevant databases in each country. Six hundred forty-three children aged 11–17 years (SD=3.4) responded to the SMFQ.

Data are available to researchers via the UK data service. As data could not be used to re-identify individuals, no further ethical approval was needed for our secondary data analysis.

### Measures

#### Short Moods and Feelings Questionnaire

Depressive symptoms were assessed with the 13-item SMFQ.^29^ Participants indicated depressive symptoms over the last 2 weeks with response categories ‘*not true’* (0), ‘*sometimes’* (1) and ‘*true’* (2), leading to scores from 0 to 26. Showing good reliability and validity, the SMFQ was designed as a depression screener in children and adolescents.^29^ Internal consistencies were good in the MHCYP (α=0.87), LAC (α=0.90) and MCS (α=0.93). Items are listed in full in table 3.

**Table 3:** Descriptive statistics of the SMFQ items for each of the three studies

	MHCYP				MCS				LAC				Comparisons			
	M	SD	Sk	KT	M	SD	Sk	KT	M	SD	Sk	KT	ANOVA	1<2	1<3	2 vs 3
I felt miserable or unhappy.	0.48	0.64	0.99	0.13	0.67	0.61	0.33	0.66	0.63	0.69	0.64	0.73	P<0.001	*	*	ns
I did not enjoy anything at all.	0.24	0.54	2.18	3.72	0.35	0.55	1.29	0.69	0.55	0.69	0.87	0.49	P<0.001	*	*	*
I felt so tired I just sat around and did nothing.	0.46	0.64	1.06	0.01	0.65	0.67	0.54	0.75	0.57	0.69	0.81	0.58	P<0.001	*	*	**
I was very restless.	0.45	0.64	1.09	0.06	0.52	0.64	0.86	0.33	0.54	0.68	0.89	0.42	P<0.001	*	**	ns
I felt I was no good anymore.	0.23	0.53	2.27	4.15	0.36	0.62	1.50	1.08	0.35	0.64	1.61	1.25	P<0.001	*	*	ns
I cried a lot.	0.21	0.49	2.35	4.75	0.33	0.60	1.64	1.53	0.36	0.65	1.57	1.11	P<0.001	*	*	ns
I found it hard to think properly or concentrate.	0.44	0.60	1.05	0.07	0.64	0.68	0.62	0.74	0.56	0.66	0.76	0.52	P<0.001	*	*	***
I hated myself.	0.19	0.49	2.52	5.53	0.32	0.59	1.73	1.83	0.34	0.63	1.62	1.34	P<0.001	*	*	ns
I felt I was a bad person.	0.17	0.44	2.58	6.10	0.25	0.51	2.00	3.13	0.35	0.62	1.57	1.25	P<0.001	*	*	***
I felt lonely.	0.28	0.54	1.78	2.21	0.43	0.65	1.22	0.27	0.45	0.68	1.18	0.09	P<0.001	*	*	ns
I thought nobody really loved me.	0.18	0.47	1.62	6.08	0.29	0.58	1.87	2.33	0.37	0.67	1.55	0.96	P<0.001	*	*	*
I thought I would never be as good as other kids.	0.31	0.56	1.61	1.62	0.41	0.65	1.34	0.54	0.52	0.71	0.97	0.40	P<0.001	*	*	*
I did everything wrong.	0.21	0.47	2.24	4.29	0.31	0.58	1.69	1.77	0.38	0.63	1.43	0.85	P<0.001	*	*	***

ANOVA with group as factor followed by post hoc comparisons using the Tukey correction.

*P<0.05; **p<0.01; ***p<0.001.

ANOVA, analysis of variance; KT, kurtosis; LAC, looked after by local authorities; MCS, Millennium Cohort Study; MHCYP, Mental Health of Children and Young People in Great Britain; ns, not significant; Sk, skewness; SMFQ, short Moods and Feelings Questionnaire.

### Analysis procedure

Analyses were performed in R.^30^ Given that items had three response options, they were treated as ordinal. We computed a separate Gaussian Graphical Model (GGM) for the SMFQ in each of the samples. A GGM consists of nodes that constitute symptoms, which are connected by edges. These edges are the estimates of the partial correlation between pairs of nodes, after adjusting for the influence of all other nodes in the network.^12^ For the three cross-sectional networks, we applied the least absolute shrinkage and selection operator. A regularisation term ensures only the most robust associations between nodes appear in the networks.^31^ We compared the overall connectivity of the three networks with the network comparison test (NCT).^31^ For each symptom, we estimated the expected influence centrality,^32^ a measure of a node’s interconnectedness with other nodes (ie, sum of edge weights connected to a node). This way, we aimed to quantify the importance of a symptom. Further analyses estimated network accuracy and stability (online supplemental 1). The edge weight difference test indicates whether specific symptom connections (ie, edges) are significantly different from other symptom connections. Similarly, the centrality difference test quantifies whether some symptoms are significantly more central in the networks than others. More detailed information on the analytical details of fitting the networks is provided in online supplemental material 1.

10.1136/bmjment-2023-300707.supp1 Supplementary data

## Findings

Samples had comparable demographics except as follows. MHCYP participants were younger than MCS participants; compared with both other samples, looked-after children were more likely male, and MCS more likely non-white (table 2). Most looked-after children lived with foster parents, but the sample included children living in children’s home and secure units. MHCYP had lower SMFQ item response means than the other two samples (table 3). Item differences were mixed when comparing the LAC sample with MCS: tiredness and concentration problems were higher in MCS whereas anhedonia and negative self-evaluations (*bad person, nobody loved me, not as good as others, did everything wrong*) were higher in the LAC sample. Items exhibited skew (≤2.58 for all items) and kurtosis (≤6.10 for all items) (see table 2). Histograms of all items can be found in online supplemental figures s1-s3. Accuracy plots showed small to moderate CIs, indicating edge weight stability (online supplemental figures s4-s6). Case-drop bootstrapping results indicated strong stability of expected influence centrality measures (online supplemental figures s7-s9). Finally, the correlation stability coefficient was ≥0.82 for all networks, indicating high stability of the estimates.

**Figure 1:**
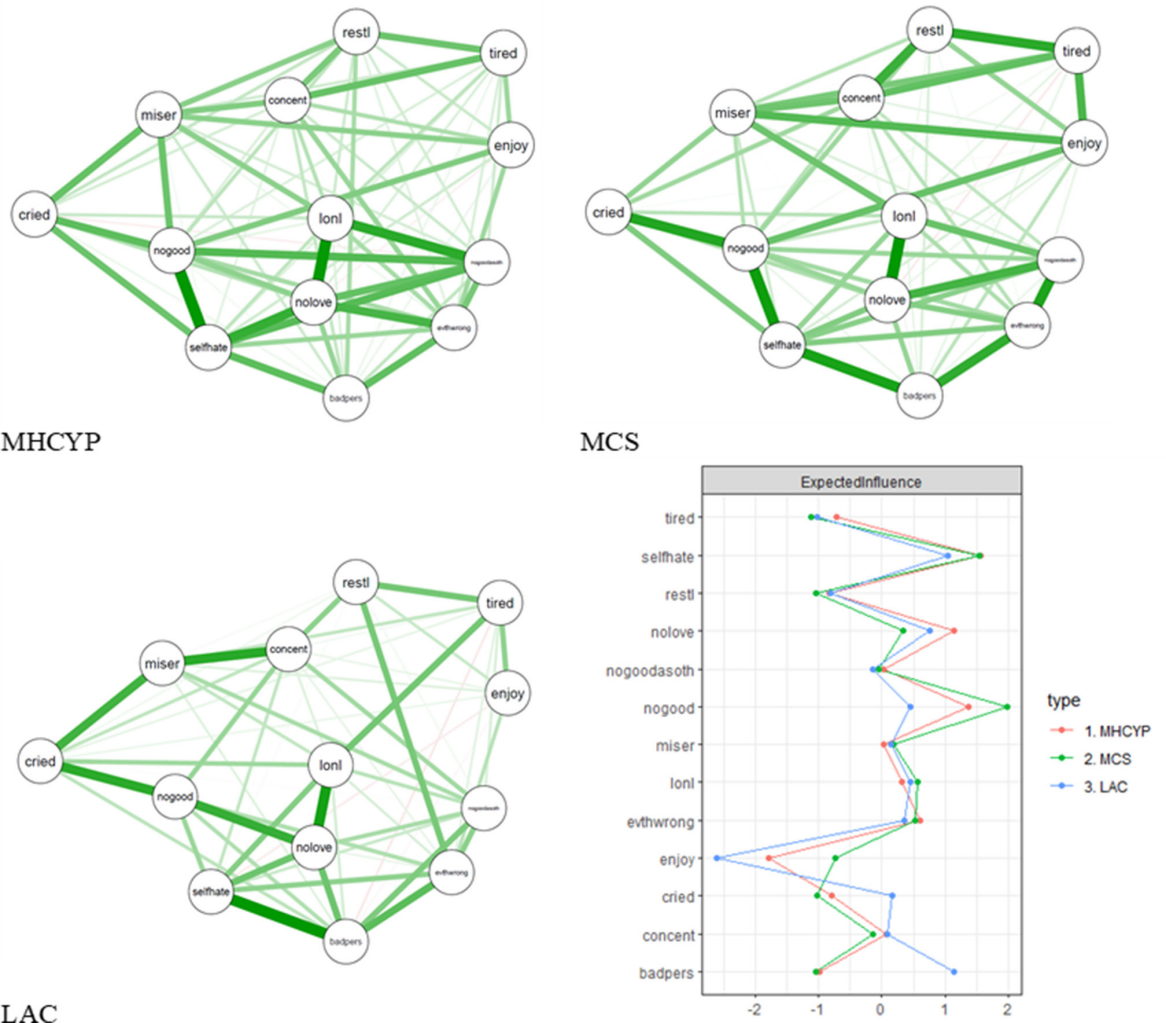
Cross-sectional networks for the three studies along with the centrality plot for these networks. We used an average layout across all three cross-sectional networks. This means that nodes of all three networks were placed at the same position so that the networks can be more easily compared visually. Nodes represent symptoms and arrows represent estimates of partial correlation. The colour of the arrows represents the directionality of the effect (green=positive effect, red=negative effect). LAC, looked after by local authorities; MCS, Millennium Cohort Study; MHCYP, Mental Health of Children and Young People in Great Britain; thicker arrows indicate stronger edges within the network. Tired, felt tired; selfhate, I hated myself; restl, restless; no love, nobody really loved me; nogoodasoth, not as good as other kids; nogood, I was no good anymore; miser, miserable/unhappy; lonl, felt lonely; evthwrong, did everything wrong; enjoy, did not enjoy anything; cried, cried a lot; concent, hard to concentrate; badpers, I was a bad person.

According to the NCT, MHCYP and LAC networks did not differ significantly from each other on overall connectivity, p=0.25. The MCS network had significantly higher overall connectivity compared with MHCYP and LAC networks (both p<0.001). The edge lists (ie, rank-order of all edges in the network) of the three networks were moderately correlated, r=0.55–0.69 (see online supplemental 2-4). Figure 1 shows the strength of these symptom connections as networks. In both the MCS and MHCYP networks, the strongest respective edges were between *lonely—nobody loved me* (regularised partial correlations (r*=*0.25–0.22) and *no good anymore—hated myself* (r*=*0.24–0.23). In the LAC network, the strongest edges were between *hated myself—bad person* (r*=*0.30), followed by *lonely—nobody loved me* (r*=*0.28). The connection *no good anymore—hated myself* was not among the strongest edges in LAC (r*=*0.12) but was significantly stronger than many other edges in MCS and MHCYP (see edge weights difference plots, online supplemental figures s10-s12).

10.1136/bmjment-2023-300707.supp2 Supplementary data

10.1136/bmjment-2023-300707.supp3 Supplementary data

10.1136/bmjment-2023-300707.supp4 Supplementary data

The expected influence centrality of each symptom per sample can be found in figure 1. Importantly, centrality estimates did not correlate with item variance (p>0.18), indicating that centrality is not driven by the variance of the symptoms. In all samples, *hated myself* and *nobody loved me* emerged as central symptoms in the networks (figure 1). The symptom *no good anymore* was very central in the MHCYP and MCS networks, and to a lesser extent in the LAC network. In all samples, the items *lonely*, *miserable* and *did everything wrong* were also among the more central symptoms. In contrast, the symptom *bad person* was a central symptom in the LAC network, but was one of the least central symptoms in the other datasets. The symptom *did not enjoy anything* was among the least central symptom in all networks. These symptoms had significantly higher and lower expected influence centrality, respectively, than many other symptoms within the networks, as indicated by the expected influence centrality difference plots in online supplemental figures s13-s15. Correlations of expected influence centralities across the samples were moderate for LAC with the other two samples (r=0.48 (MCS), r=0.66 (MHCYP)) and high between the MHCYP and MCS network, r=0.91.

## Discussion

We examined depressive symptom constellations among adolescents in UK general population samples and in adolescents looked-after by local authorities. Symptoms surrounding self-hate and the perception that the adolescent was *no good anymore* were core symptoms in the two general population samples, and among the most core symptoms in LAC. These symptoms were also central in previous network studies using the SMFQ^15 17 18^ and networks studies using the CDI.^17 19–21^ These symptoms encompass feelings of worthlessness as covered in depression diagnoses in the DSM-5.^10^ The emergence of such symptoms may constitute a vicious circle. When adolescents feel that *nobody loves* them, they may conclude they are *no good anymore* or start to *hate* themselves. Alternatively, when adolescents perceive that love is conditional on being good, feeling *no good anymore* may lead to perceptions that *nobody loves* them. These central symptoms highlight the importance of self-esteem in depression,^10^ and the necessity to detect and counteract feelings of worthless as early as possible at both home and school.

In former cross-sectional network analyses using SMFQ and CDI, loneliness was consistently one of the most central symptoms from ages 11 to 19 years.^14 15 17 19 20^ In all three datasets we studied, loneliness was also among one of the most central symptoms, with important connections to core symptoms, especially the feeling that *nobody loved* the young person. Adolescence is a period of increased vulnerability to loneliness with detrimental mental health consequences.^22^ Thus, the emergence of feelings of loneliness needs to be carefully monitored and counteracted before other depressive symptoms manifest. Across networks, *not enjoying anything* was consistently one of the least central symptoms, indicating that anhedonia is not strongly influencing, or influenced by, other symptoms. This is consistent with former reports that anhedonia is more important in symptom presentations in adults (aged 18 years and above) compared with adolescents.^4^

While overall the three network structures were similar, there was one key difference between the looked-after children network compared with the general population networks. In the looked-after children, the symptom *bad person* had the highest centrality, yet this was one of the least central symptoms in the other two networks. This symptom also had higher mean levels in looked-after children. In addition, *hated myself* and *no good anymore* had strong connections in the other two datasets but a comparably weak connection among looked-after children. In looked-after children, the strongest edges were found between *hated myself and bad person*. In contrast with *no good anymore* (a key symptom across all three datasets), being *a bad person* constitutes a more holistic assessment of the adolescent’s worth. *I was no good anymore* suggests one believes their abilities or worthiness have declined, implying a perceived loss of competence or value, which may be domain specific. However, being *a bad person* represents a broader and more pervasive negative self-evaluation encompassing one’s overall character and identity. Understanding this distinction is important in clinical practice as it guides the assessment process, treatment planning and interventions aimed at modifying maladaptive cognitions. Due to life circumstances, looked-after children are more likely to experience exclusion from mainstream schools, bullying or stigmatisation than young people in the general population.^6^ Throughout these experiences and the abuse many have suffered,^5 7^ it may have been explicitly or implicitly suggested that they were *a bad person*. Indeed, children’s experiences of abuse and neglect have resulted in their global and stable attributions of being bad people.^23^ This may be exacerbated by comorbidity with conduct disorder which is more common among looked-after children and may have led to even more negative feedback.^5^ Additionally, the attachment styles of looked-after children are more likely to have been damaged, resulting in increased negative interactions with subsequent non-abusive caregivers—which may bolster the young person’s perception of being a bad person.^24^ Looked-after children show less secure attachment and more insecure and disorganised attachment compared with the general population.^24 26^ Stable, loving, caring environments and psychotherapy are crucial to help counteract such attachment styles and perceptions in looked-after children.^5 7^ Indeed, stable care environments can help restore secure attachment in adopted children.^25^

The overall connectivity of the MCS network was somewhat higher compared with the other two networks, which in theory suggests that this network is more easily activated by a single symptom than the other networks.^13^ This could reflect secular increases in mental health problems over time or differences based on demographic characteristics, as the MCS had more non-white participants.^20^ However, the network structure of the two population-based studies was similar (eg, central symptoms and edge weights), aligning with recent population-based networks studies of the SMFQ.^15 17 18^

We also found a lower proportion of significantly different edge weights and expected influence centrality scores for the LAC sample compared with the other samples, possibly due to a lower sample size. Future larger samples of looked-after children could determine whether this reflects fewer core symptoms and more homogeneity in symptom correlations in looked-after children. Such a finding could mean that symptoms are equally important in looked-after children, making it difficult to discern which ones to target. Or, if fewer are central, then it may be easier to target key symptoms.

### Clinical implications

Theoretically, targeting core symptoms in a network should reduce overall network connectivity, but this is yet to be examined.^13^ Nonetheless, our findings indicate a different symptom profile in adolescents versus adults, where *sadness* and *loss of interest* are core symptoms,^11^ which we did not observe. Our central symptoms identified across three samples were more consistent than findings from other adolescent studies (table 1). Our findings provide impetus for targeting symptoms of self-worth in adolescent depression intervention studies, to test whether alleviating these symptoms aids recovery from depression. In looked-after children, additionally targeting *feeling like a bad person* appears important, although our network findings would benefit from replication prior to testing this in a clinical setting.

### Strengths and limitations

This study used the same analysis procedure and measure across three different samples which varied in their population representativeness in terms of their experienced adversities. The large sample sizes allowed for a detailed network analytical approach. However, our analyses were cross-sectional. Longitudinal network studies help elucidate the temporal role of symptom centrality,^13^ highlighting symptoms which subsequently influence other symptoms, and thus may be more meaningful for intervention. Furthermore, significant associations between symptoms may not be clinically meaningful.^13^ Future research should therefore assess the relevance of our findings in clinical populations. The MCS data are more recent than the other two datasets, and mean symptom levels of the other population-based study (MHCYP) were significantly lower, suggesting a possible secular increase in levels of depressive symptoms over time. The MHCYP and LAC datasets were collected within a year of each other, and thus the higher symptoms observed in LAC appear indicative of greater symptomatology in this vulnerable population. As the key differences in the looked-after children’s networks were comparable for both population-based datasets, this seems to reflect genuine differences instead of being an artefact of different time lags between studies. Furthermore, demographics differed between samples. While network structures can differ based on ethnicity,^20^ this is unlikely to have been influential in our study, as the network structures of the MHCYP and MCS were similar despite MCS having more non-white participants. Similarly, age differences between these two samples did not appear to have a role in driving network differences, although the further age differences in LAC may have contributed to the network differences observed in this study. Finally, as LAC participants were more likely to be male, this may have influenced results if boys for instance are more likely to conclude that they are a bad person. Future studies should also examine potential differences in the network structure based on specific care arrangements, for which we lacked power in the present study.

## Conclusion

This research sheds light on depressive symptom inter-relations in adolescents. Across three studies, *self-hate* and *I was no good anymore* emerged as key symptoms, aligning with prior work but more clearly underscoring the importance of these symptoms. In looked-after children, *I was a bad person* had the highest centrality, yet this was one of the least central symptoms in the other datasets. Thus, negative self-evaluation may exert a more significant impact on looked-after children’s depressive symptoms than the general population.

## Data Availability

All data are available to researchers via the UK data service.
