# Supplementary material for: Depressive symptom networks in the UK general adolescent population and in those looked after by local authorities
Source: BMJ Ment Health. 2023 Sep 1;26(1):e300707. doi: 10.1136/bmjment-2023-300707 (PMC10577707; doi:10.1136/bmjment-2023-300707)
Supplement: Supplementary data [file bmjment-2023-300707supp001.pdf]

BMJ Publishing Group Limited (BMJ) disclaims all liability and responsibility arising from any reliance placed on this supplemental material which has been supplied by the author(s)

Supplemental Material for

Depressive symptom networks during adolescence in the general population and looked-after adolescents

Supplemental Material :

Full Analysis Procedure

Analyses were performed with R version 4.01 (1). We provide all information to enable independent replication of our results (2). Given the small amount of missing data on the SMFQ (<5% on single items), we treated data as missing at random. The missing data points were imputed with the R package *mice* (3). We computed a separate Gaussian Graphical Model (GGM) for the SMFQ in each of the samples using the *estimateNetwork* function in the *bootnet* package with the cor_auto function from the qgraph package. This function selects the correlation method based on the nature of the data, so polychoric correlations were used for the present ordinal data (4). A GGM consists of nodes that constitute symptoms, which are connected by edges. These edges are the estimates of the partial correlation between pairs of nodes, after adjusting for the influence of all other nodes in the network (5). We visualized the networks with the R package *qgraph* (4). For the three cross-sectional networks, we applied the least absolute shrinkage and selection operator with model selection using Extended Bayesian Information Criterion (EBIC) and the tuning parameter gamma set to the default value of 0.5 (6). This estimator avoids the estimation of spurious connections with a regularization term. Accordingly, only the most robust associations between nodes appear in the networks (4). We compared the overall connectivity of the three networks with the network comparison test (NCT) (4). For each symptom, we estimated the expected influence centrality, which is a measure of a node's interconnectedness with other nodes (i.e., the sum of the edge weights connected to a node) (7). This way, we aimed to quantify the importance of a symptom (7). To estimate the accuracy of edge weights, we placed 95% confidence intervals (CIs) around each edge weight; the CIs were constructed using nonparametric bootstrapping with 1,000 iterations. Using the *bootnet* package, we calculated the correlation-stability, where the centrality indices from the entire sample are correlated with centrality indices estimates on subsets of the sample (range: 0 to 1, case-drop

bootstrapping, (4)). We also report the correlation stability coefficient, which represents the stability of findings by determining the maximum number of cases that can be removed from the data while ensuring a correlation of at least 0.7 between statistics calculated based on the original network and statistics calculated with a reduced number of cases. It is recommended that this coefficient should be higher than 0.5 (4). The edge weight difference test and centrality difference test were used to examine whether edges and centrality indices differ significantly from each other [ibid]. The former test indicates whether specific symptom connections (i.e., edges) are more important than other symptom connections. Similarly, the latter test quantifies whether some symptoms are more important (i.e., central) in the networks than other symptoms.

1. R Core Team. R: A language and environment for statistical computing. R Foundation for Statistical Computing, Vienna, Austria. 2012. 2021.

2. Burger J, Isvoranu AM, Lunansky G, Haslbeck J, Epskamp S, Hoekstra RH, u. a. Reporting standards for psychological network analyses in cross-sectional data. Psychol Methods. 2022;

3. van Buuren S, Groothuis-Oudshoorn K, Robitzsch A, Vink G, Doove L, Jolani S. Package 'mice'. Comput Softw. 2015;

4. Epskamp S, Borsboom D, Fried EI. Estimating psychological networks and their accuracy: A tutorial paper. Behav Res Methods. 1. Februar 2018;50(1):195–212.

5. Borsboom D, Cramer AO. Network analysis: an integrative approach to the structure of psychopathology. Annu Rev Clin Psychol. 2013;9(1):91–121.

6. Foygel R, Drton M. Extended Bayesian information criteria for Gaussian graphical models. Adv Neural Inf Process Syst. 2010;23.

7. Robinaugh DJ, Millner AJ, McNally RJ. Identifying highly influential nodes in the complicated grief network. J Abnorm Psychol. 2016;125(6):747.

Figure s1. Histogram of all items of the MHCYP sample.

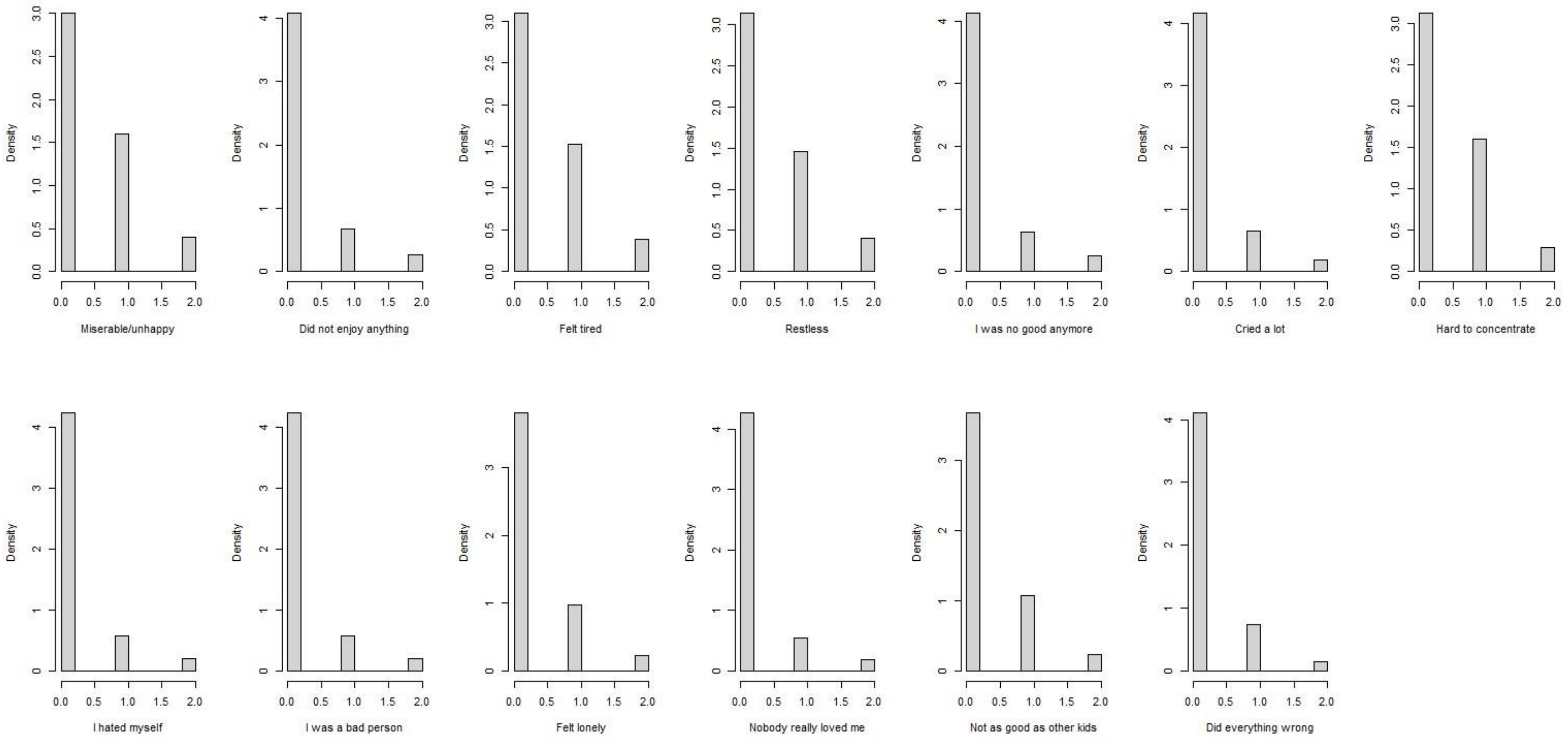

Figure s2. Histogram of all items of the MCS sample.

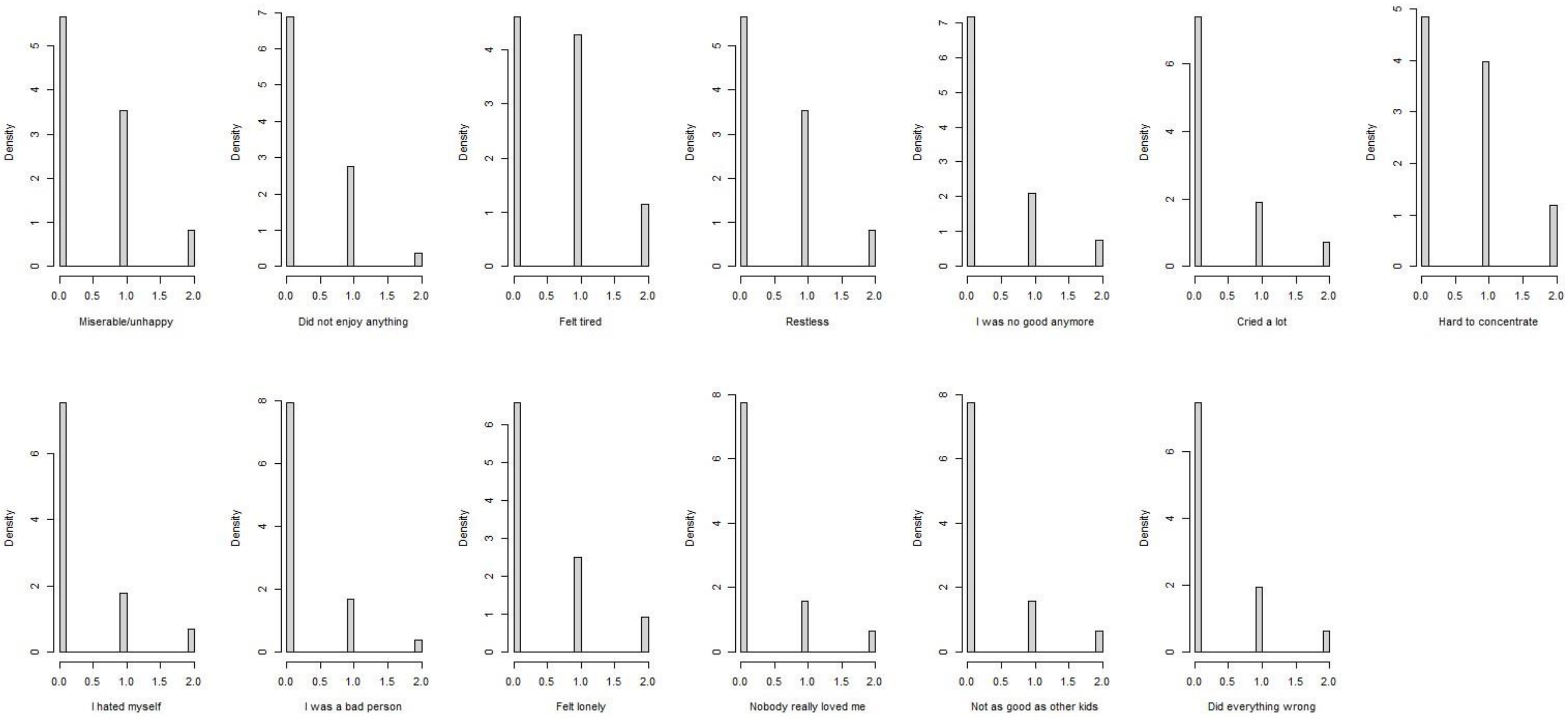

Figure s3. Histogram of all items of the LAC sample.

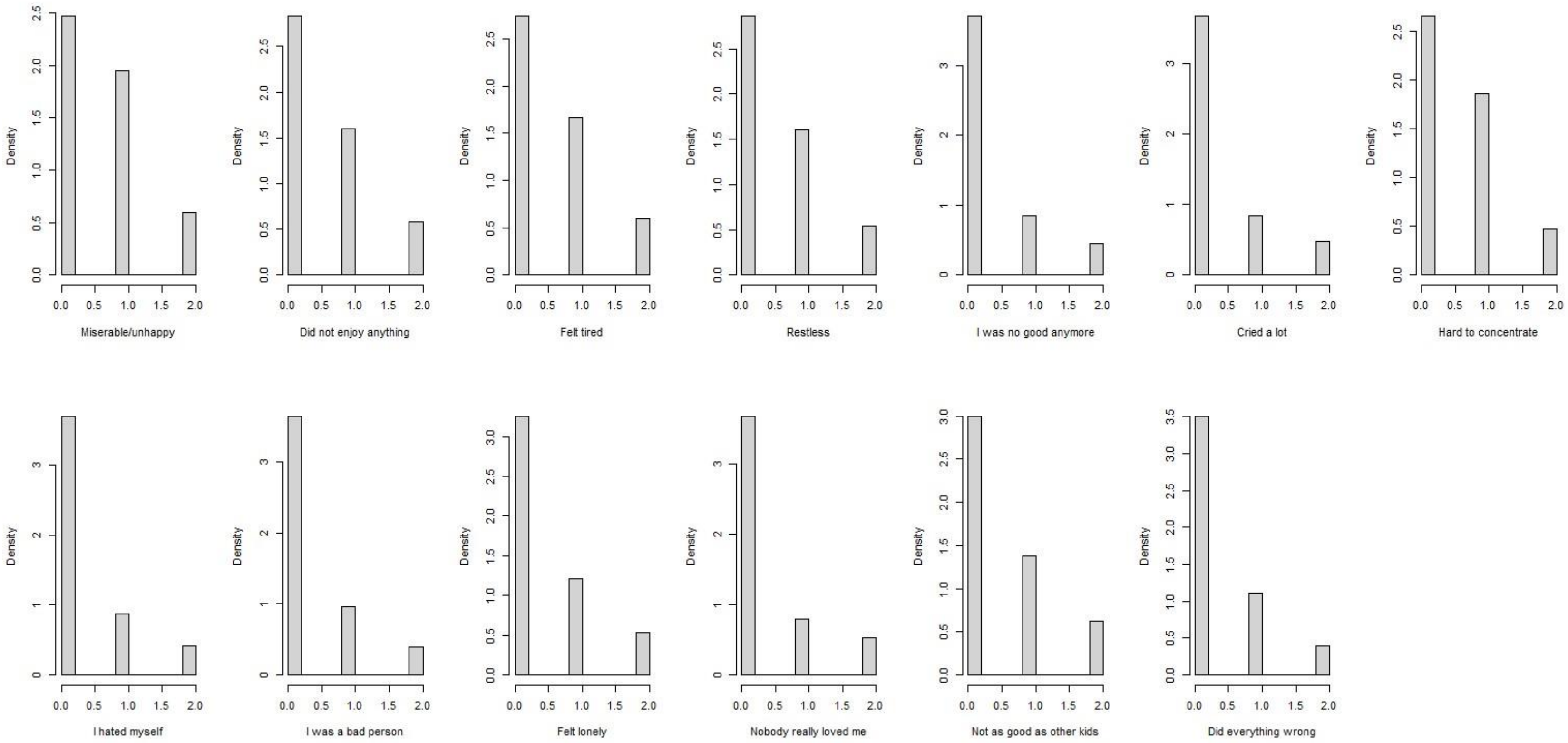

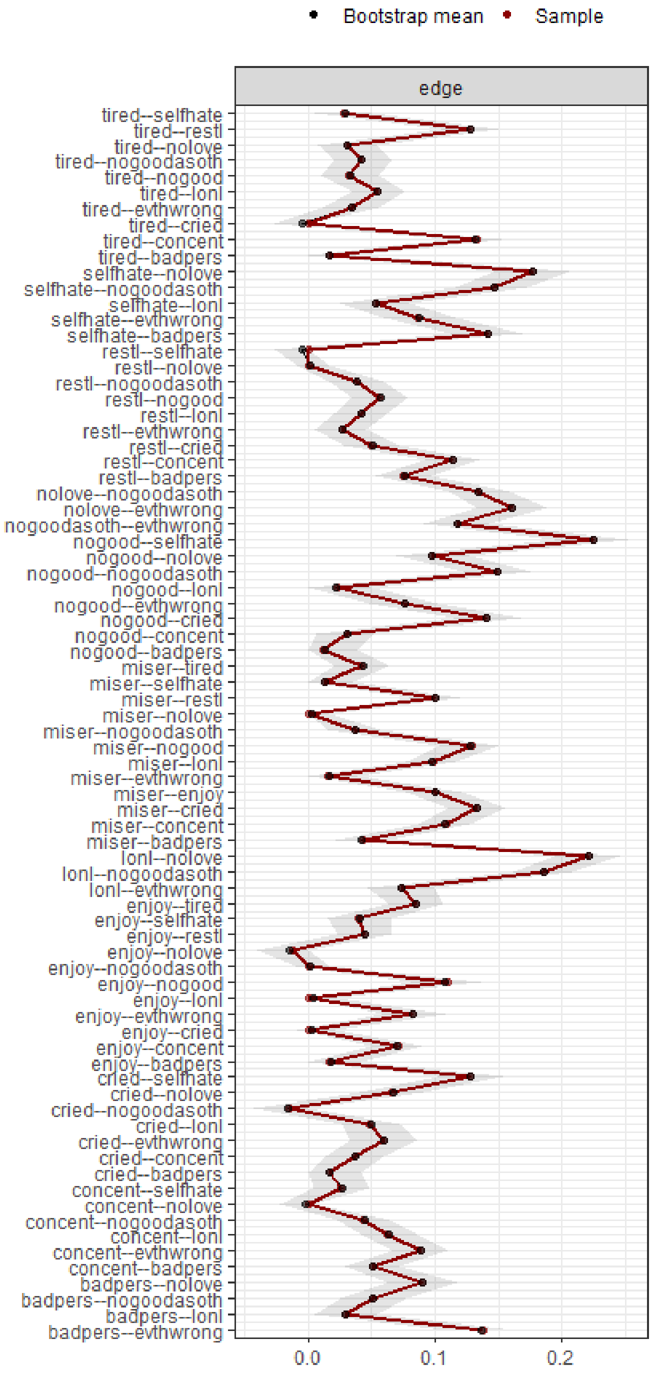

Figure s4. SMFQ interrelation accuracy plots with 1000 bootstrap iterations for the cross-sectional MHCYP network. Plots show the sample interrelations (i.e., edge weights) with the red dots, the means of the bootstrapped interrelations (i.e., edge weights) with black dots, and the bootstrap confidence intervals.

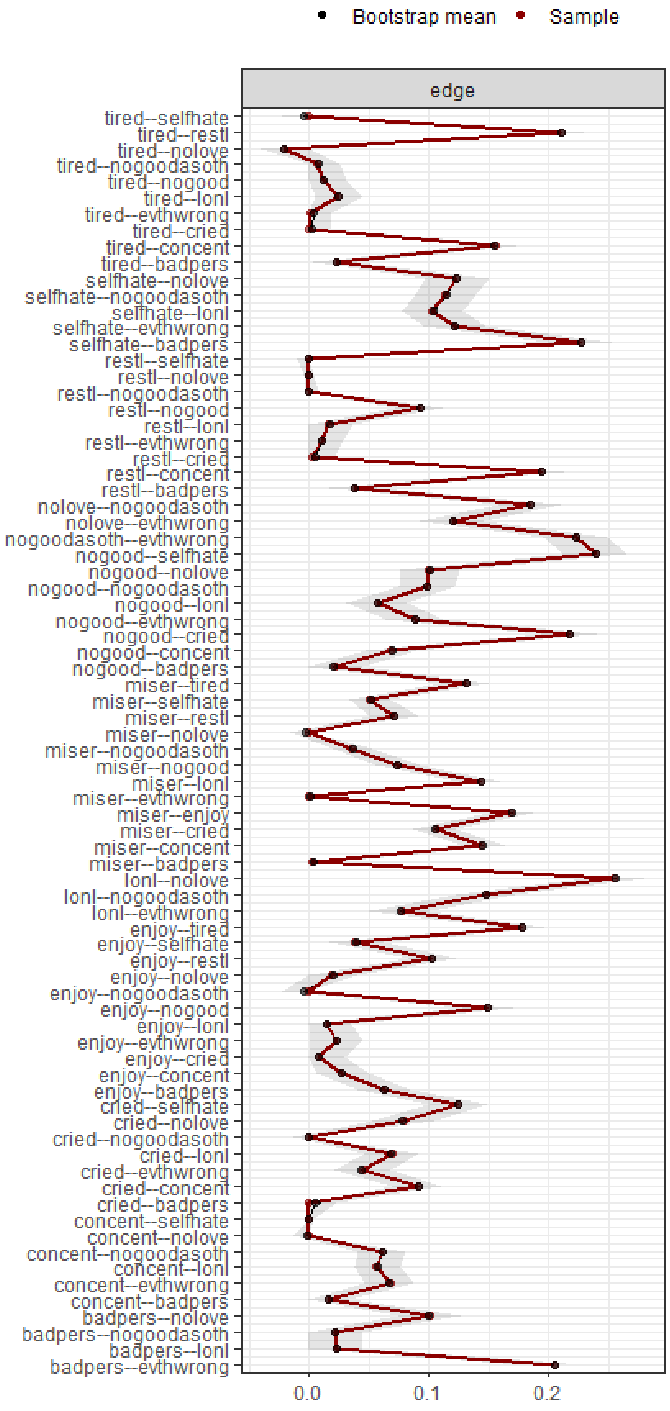

Figure s5. SMFQ interrelation accuracy plots with 1000 bootstrap iterations for the cross-sectional MCS network. Plots show the sample interrelations (i.e., edge weights) with the red dots, the means of the bootstrapped interrelations (i.e., edge weights) with black dots, and the bootstrap confidence intervals.

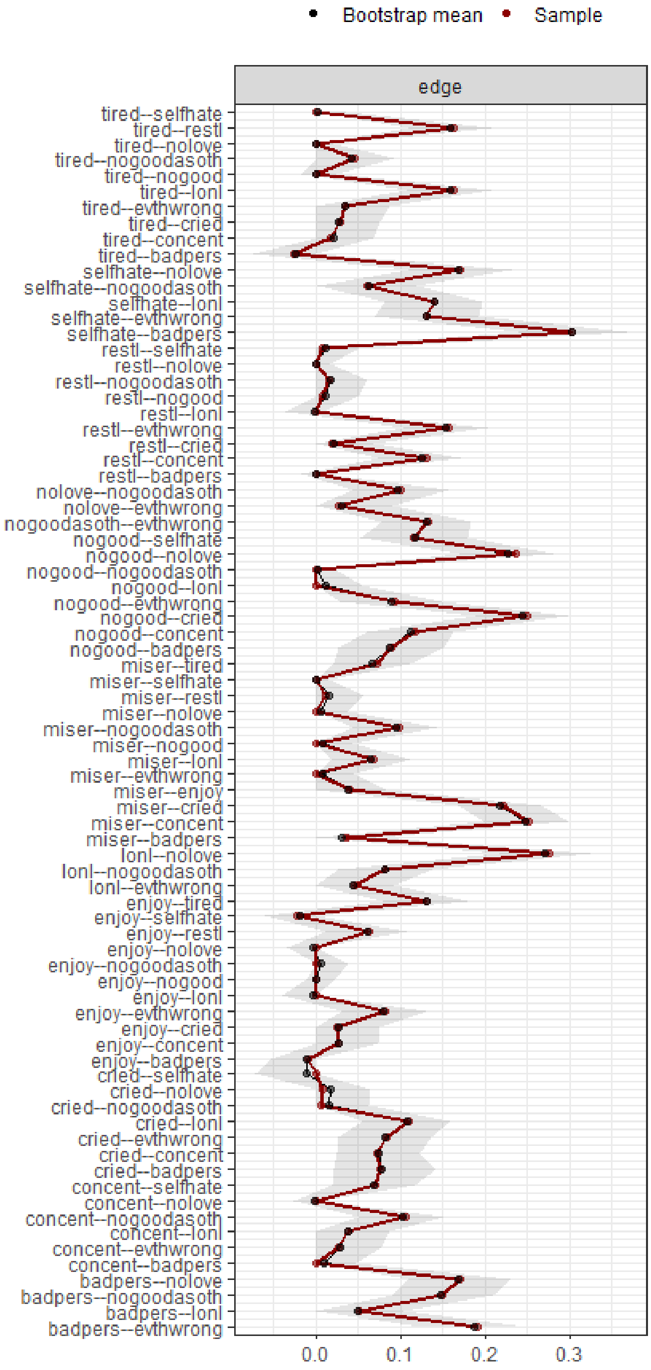

Figure s6. SMFQ interrelation accuracy plots with 1000 bootstrap iterations for the cross-sectional LAC network. Plots show the sample interrelations (i.e., edge weights) with the red dots, the means of the bootstrapped interrelations (i.e., edge weights) with black dots, and the bootstrap confidence intervals.

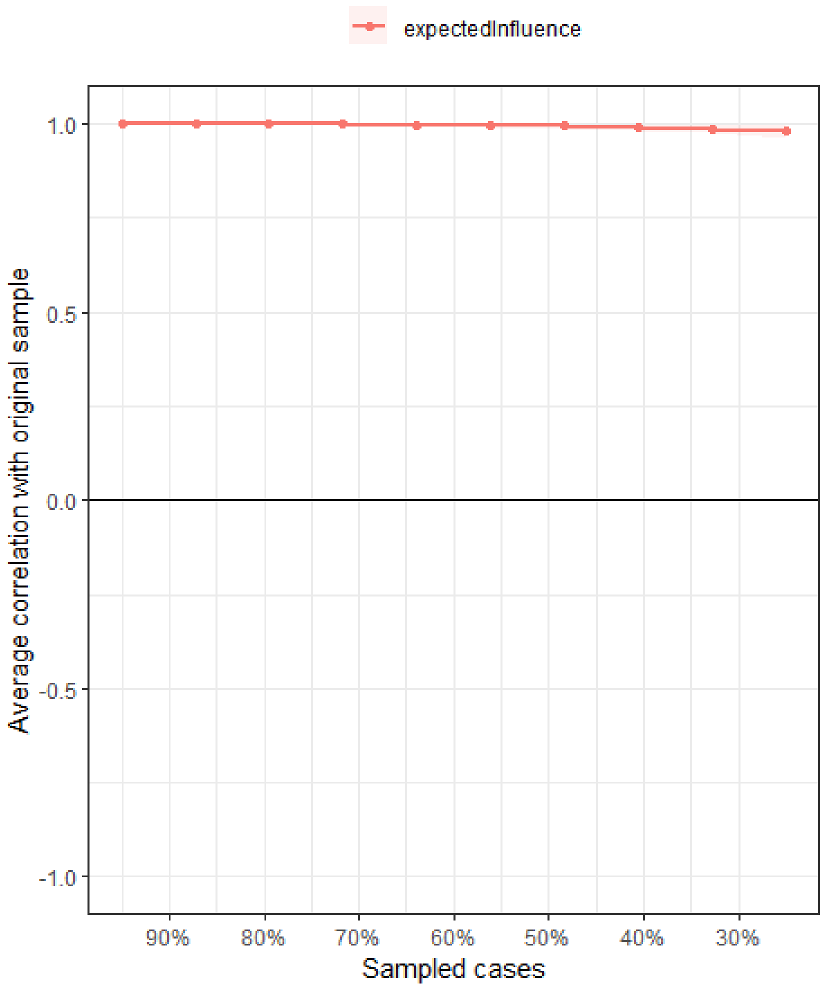

Figure s7. Stability of the Expected influence centrality measures for the MHCYP network.

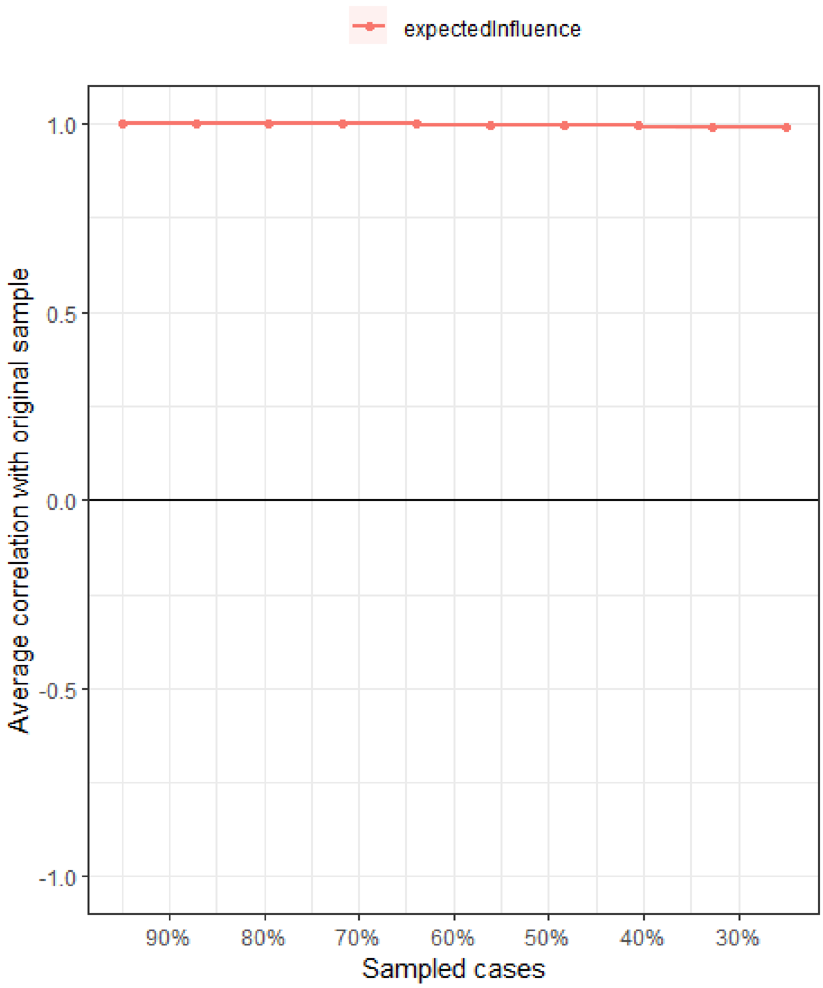

Figure s8. Stability of the Expected influence centrality measures for the MCS network.

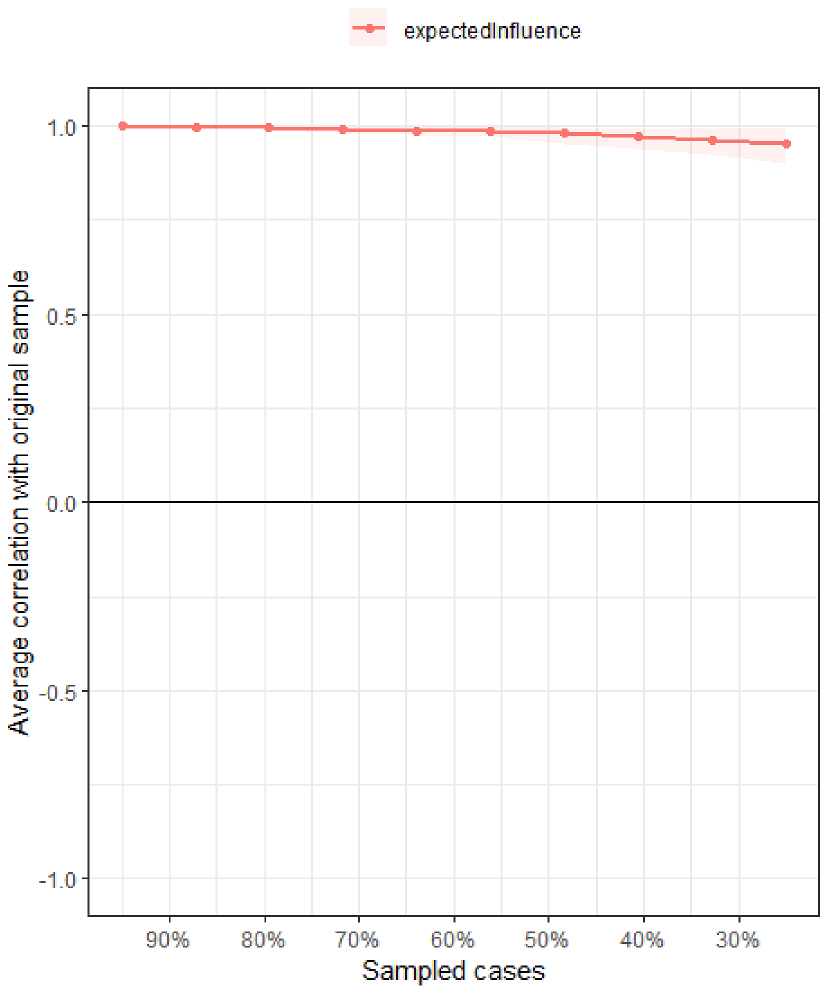

Figure s9. Stability of the Expected influence centrality measures for the LAC network.

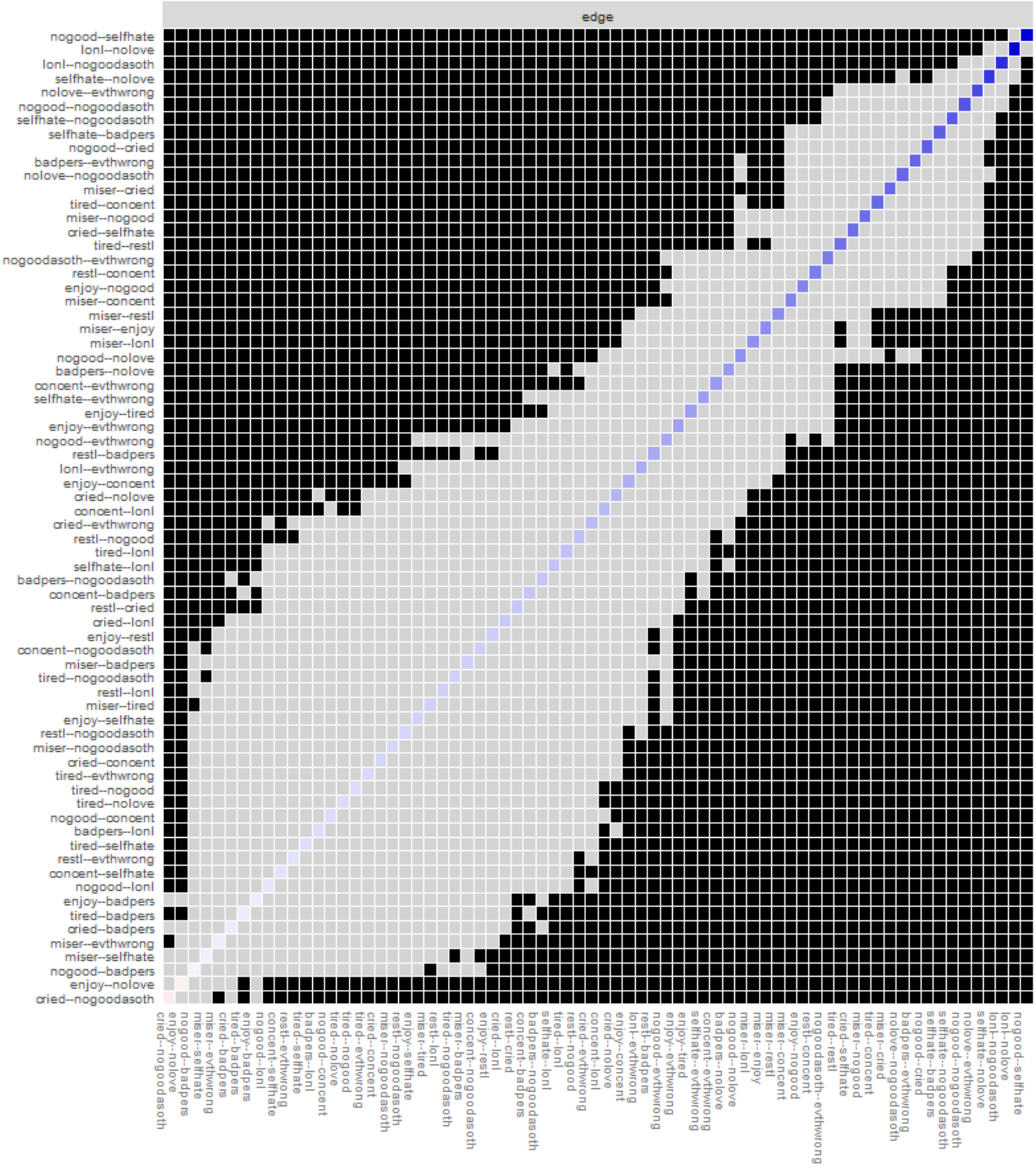

Figure s10. Edge weight difference tests for the MHCYP network. Black boxes indicate edges that significantly differ from each other ($p < .05$). Gray boxes indicate no differences. Diagonal indicates the magnitude of the associations: blue (upper right-hand corner) indicates a positive correlation, white indicates a correlation of zero, while pink (lower left-hand corner) indicates a negative correlation. Correlations are ordered by the magnitude of their association, with darker shades indicating stronger associations compared with lighter shades (the coefficients can be found in the edge list in Supplemental Material 2).

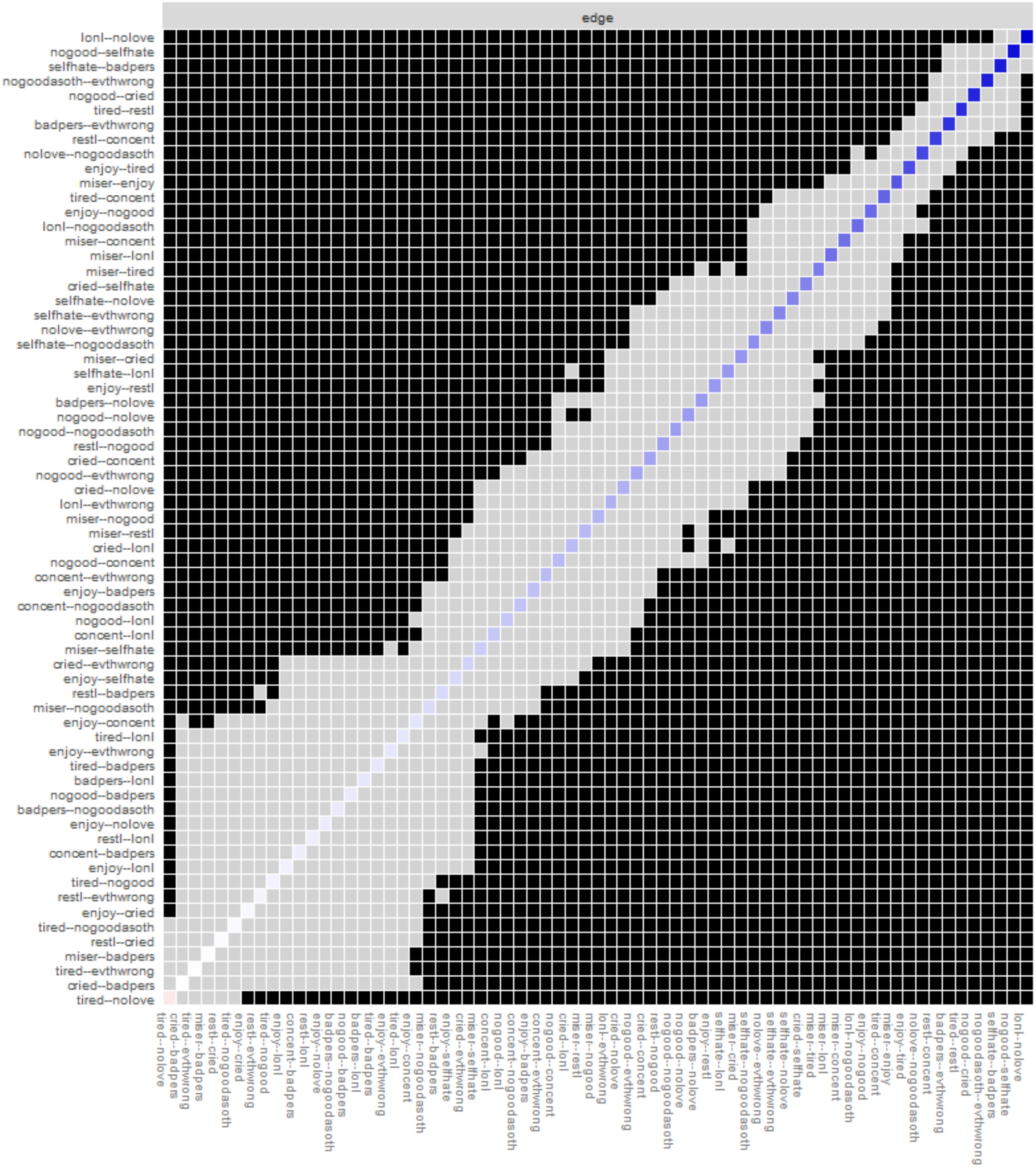

Figure s11. Edge weight difference tests for the MCS network. Black boxes indicate edges that significantly differ from each other ($p < .05$). Gray boxes indicate no differences. Diagonal indicates the magnitude of the associations: blue (upper right-hand corner) indicates a positive correlation, white indicates a correlation of zero, while pink (lower left-hand corner) indicates a negative correlation. Correlations are ordered by the magnitude of their association with darker shades indicating stronger associations compared with lighter shades (the coefficients can be found in the edge list in Supplemental Material 3).

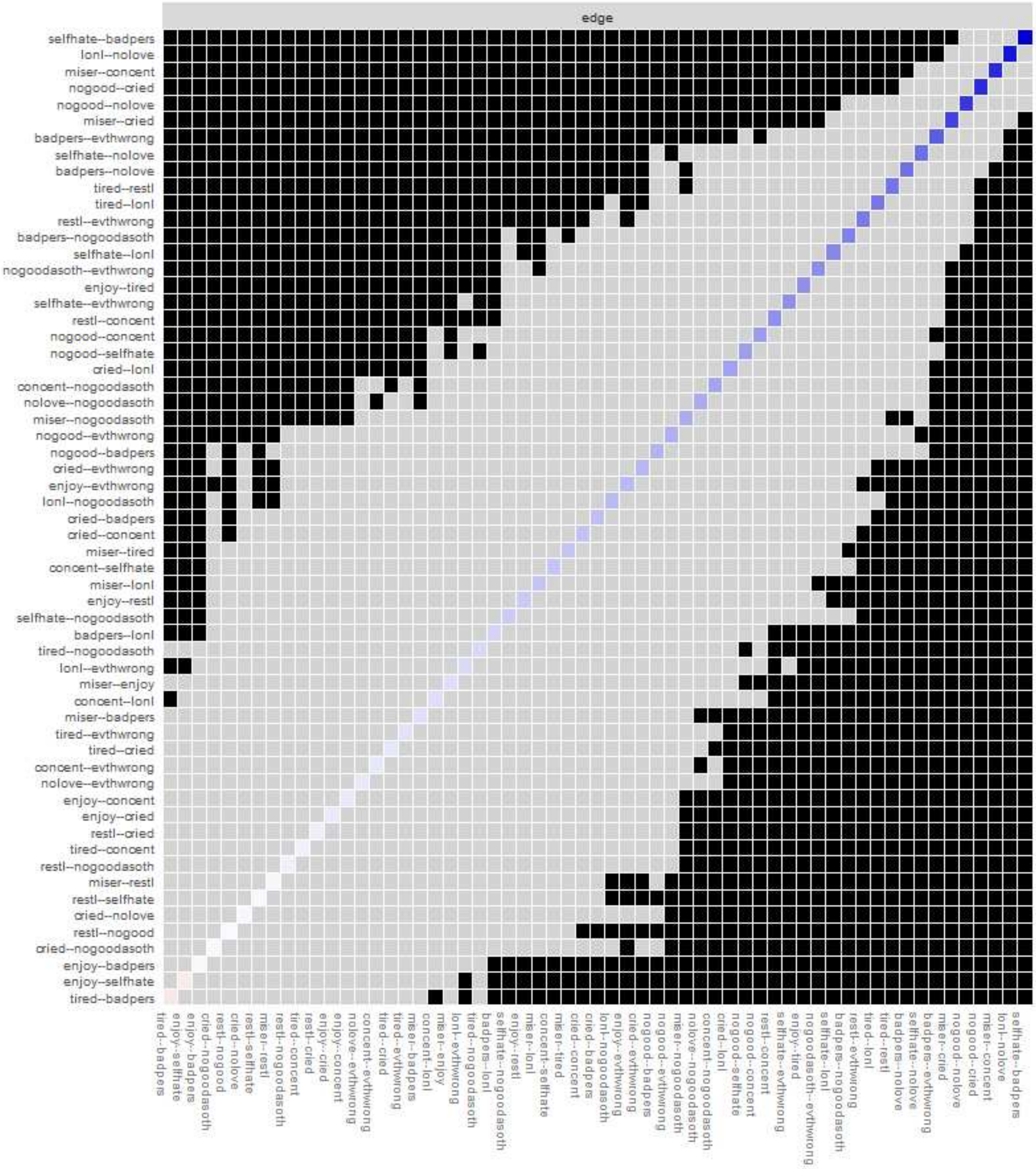

Figure s12. Edge weight difference tests for the LAC network. Black boxes indicate edges that significantly differ from each other ($p < .05$). Gray boxes indicate no differences. Diagonal indicates the magnitude of the associations: blue (upper right-hand corner) indicates a positive correlation, white indicates a correlation of zero, while pink (lower left-hand corner) indicates a negative correlation. Correlations are ordered by the magnitude of their association with darker shades indicating stronger associations compared with lighter shades (the coefficients can be found in the edge list in Supplemental Material 4).

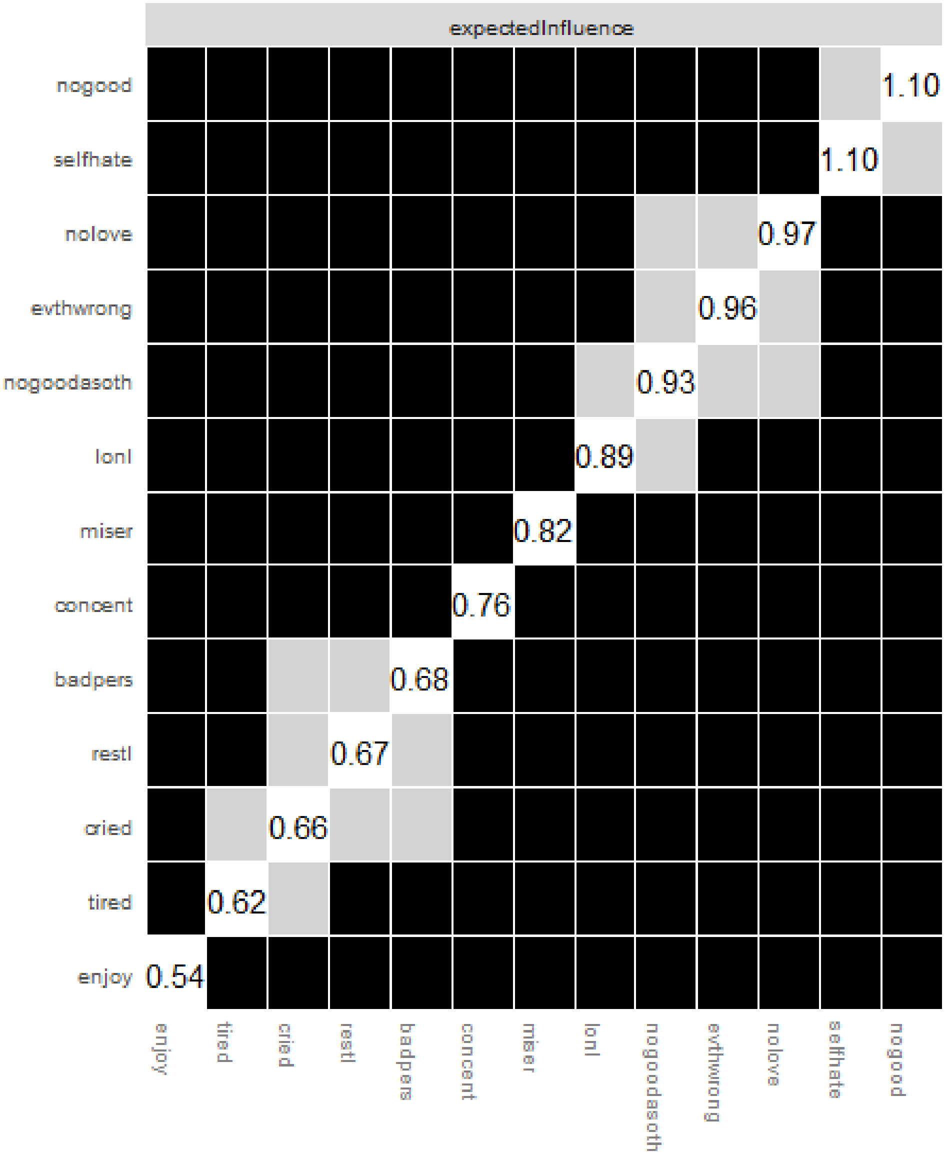

Figure s13. Expected influence difference tests for the Mental Health of Children and Young People in Great Britain network. Black boxes indicate nodes that significantly differ from each other ($p < .05$). Gray boxes indicate no differences.

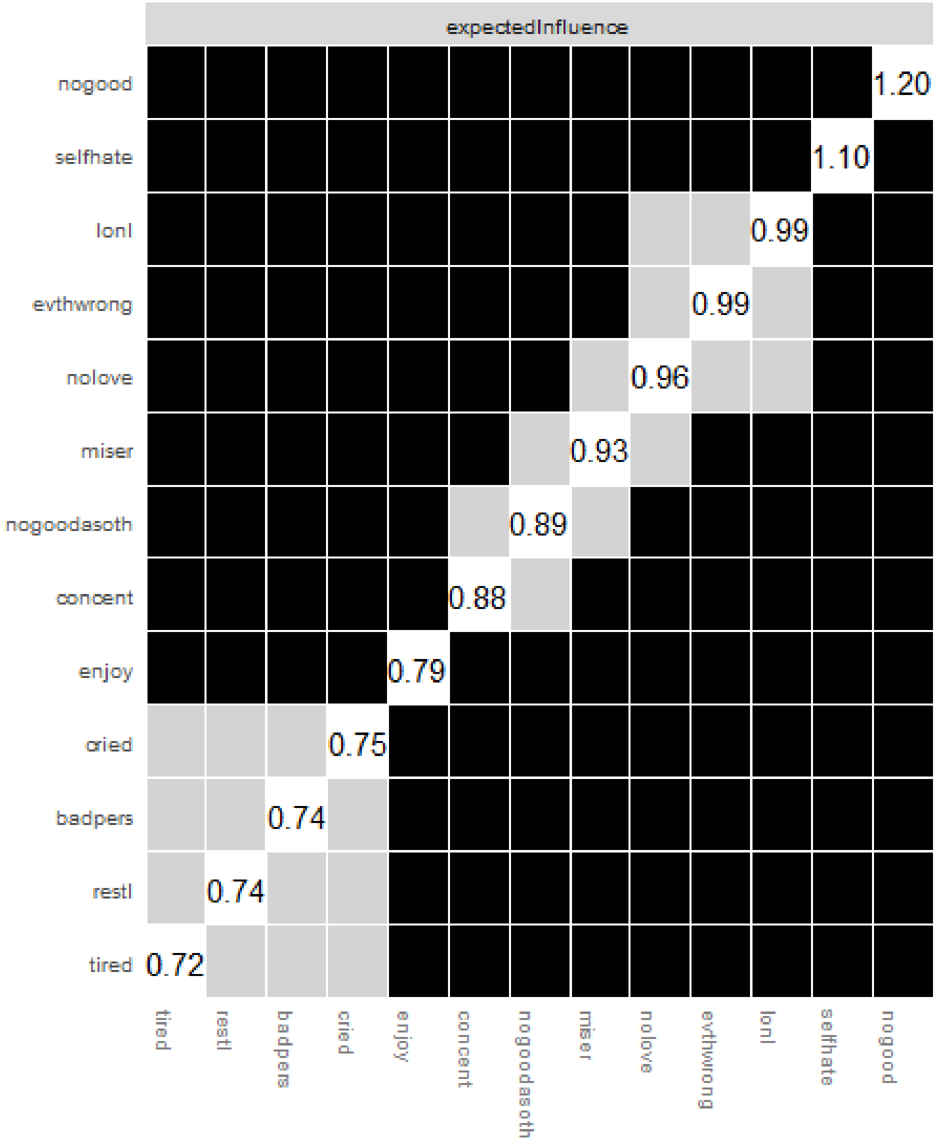

Figure s14. Expected influence difference tests for the Millennium Cohort Study People in Great Britain network. Black boxes indicate nodes that significantly differ from each other (*p* < .05). Gray boxes indicate no differences.

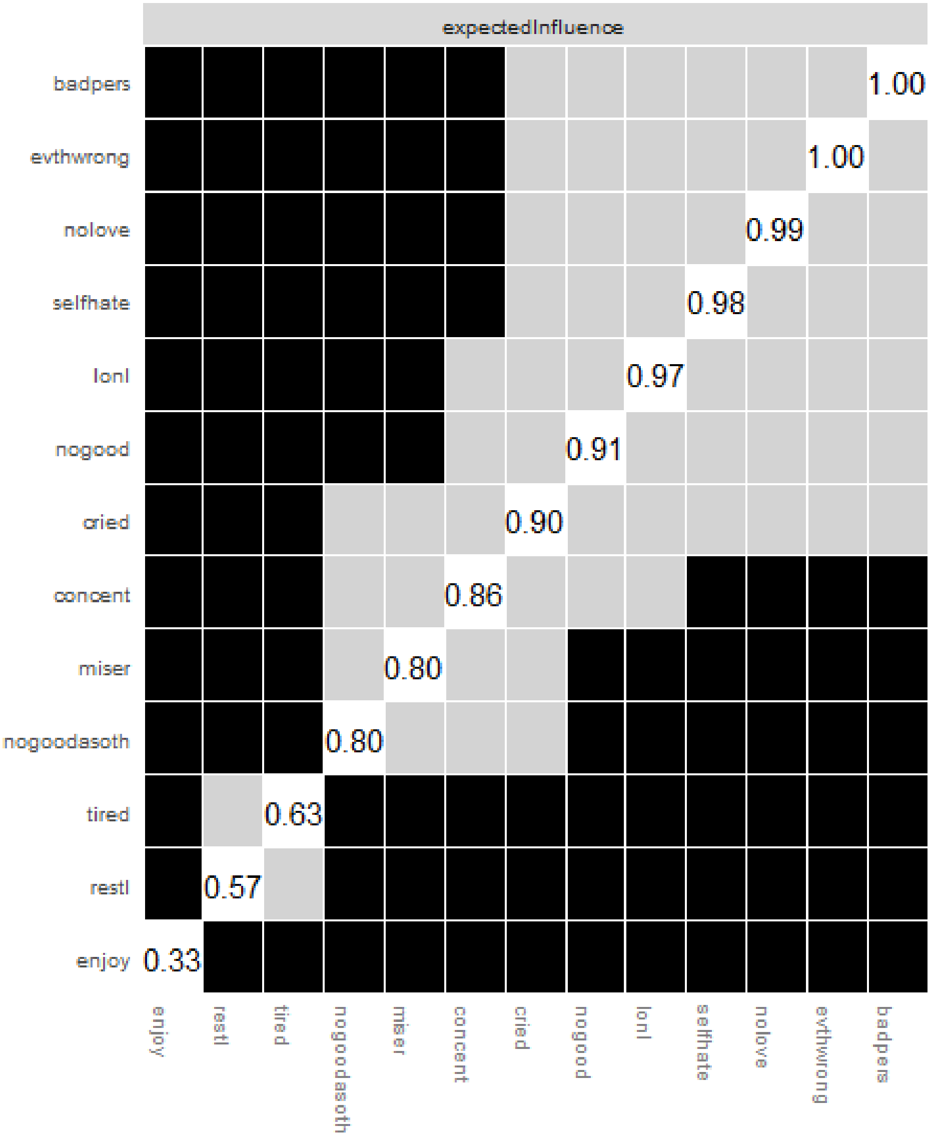

Figure s15. Expected influence difference tests for the LAC network. Black boxes indicate nodes that significantly differ from each other ($p < .05$). Gray boxes indicate no differences.
