# Supplementary material for: Depressive symptom networks in the UK general adolescent population and in those looked after by local authorities
Source: BMJ Ment Health. 2023 Sep 1;26(1):e300707. doi: 10.1136/bmjment-2023-300707 (PMC10577707; doi:10.1136/bmjment-2023-300707)
Supplement: Supplementary data [file bmjment-2023-300707supp002.pdf]

| | name | type | node1 | node2 | value | id |
|---|---|---|---|---|---|---|
| 26 | sample | edge | nogood | selfhate | 0,22480989 | nogood--selfha |
| 55 | sample | edge | lonl | nolove | 0,22118573 | lonl--nolove |
| 65 | sample | edge | lonl | nogoodasoth | 0,18611845 | lonl--nogooda |
| 53 | sample | edge | selfhate | nolove | 0,17724863 | selfhate--nolov |
| 77 | sample | edge | nolove | evthwrong | 0,16072755 | nolove--evthw |
| 60 | sample | edge | nogood | nogoodasoth | 0,1497053 | nogood--nogo |
| 63 | sample | edge | selfhate | nogoodasoth | 0,14668112 | selfhate--nogo |
| 36 | sample | edge | selfhate | badpers | 0,14189496 | selfhate--badp |
| 15 | sample | edge | nogood | cried | 0,14080724 | nogood--cried |
| 75 | sample | edge | badpers | evthwrong | 0,13775396 | badpers--evth |
| 66 | sample | edge | nolove | nogoodasoth | 0,1345642 | nolove--nogoo |
| 11 | sample | edge | miser | cried | 0,13306731 | miser--cried |
| 18 | sample | edge | tired | concent | 0,13227444 | tired--concent |
| 7 | sample | edge | miser | nogood | 0,12890404 | miser--nogooc |
| 27 | sample | edge | cried | selfhate | 0,12803168 | cried--selfhate |
| 6 | sample | edge | tired | restl | 0,12739017 | tired--restl |
| 78 | sample | edge | nogoodasoth | evthwrong | 0,11730272 | nogoodasoth-- |
| 19 | sample | edge | restl | concent | 0,11403833 | restl--concent |
| 8 | sample | edge | enjoy | nogood | 0,10972439 | enjoy--nogooc |
| 16 | sample | edge | miser | concent | 0,10825946 | miser--concen |
| 4 | sample | edge | miser | restl | 0,10033869 | miser--restl |
| 1 | sample | edge | miser | enjoy | 0,10001212 | miser--enjoy |
| 37 | sample | edge | miser | lonl | 0,09885887 | miser--lonl |
| 50 | sample | edge | nogood | nolove | 0,09769992 | nogood--nolov |
| 54 | sample | edge | badpers | nolove | 0,08998731 | badpers--nolo |
| 73 | sample | edge | concent | evthwrong | 0,08850963 | concent--evth |
| 74 | sample | edge | selfhate | evthwrong | 0,08649174 | selfhate--evth |
| 3 | sample | edge | enjoy | tired | 0,08520477 | enjoy--tired |
| 68 | sample | edge | enjoy | evthwrong | 0,08282756 | enjoy--evthwr |
| 71 | sample | edge | nogood | evthwrong | 0,07616223 | nogood--evthv |
| 32 | sample | edge | restl | badpers | 0,07512444 | restl--badpers |
| 76 | sample | edge | lonl | evthwrong | 0,0737836 | lonl--evthwror |
| 17 | sample | edge | enjoy | concent | 0,07044662 | enjoy--concen |
| 51 | sample | edge | cried | nolove | 0,0661843 | cried--nolove |
| 43 | sample | edge | concent | lonl | 0,06254894 | concent--lonl |
| 72 | sample | edge | cried | evthwrong | 0,05952991 | cried--evthwro |
| 10 | sample | edge | restl | nogood | 0,05631535 | restl--nogood |
| 39 | sample | edge | tired | lonl | 0,05433273 | tired--lonl |
| 44 | sample | edge | selfhate | lonl | 0,05334935 | selfhate--lonl |
| 64 | sample | edge | badpers | nogoodasoth | 0,05076705 | badpers--nogo |
| 35 | sample | edge | concent | badpers | 0,05039298 | concent--badp |
| 14 | sample | edge | restl | cried | 0,04978316 | restl--cried |
| 42 | sample | edge | cried | lonl | 0,04784812 | cried--lonl |
| 5 | sample | edge | enjoy | restl | 0,04396889 | enjoy--restl |
| 62 | sample | edge | concent | nogoodasoth | 0,04324441 | concent--nogo |
| 29 | sample | edge | miser | badpers | 0,0427038 | miser--badper |
| 58 | sample | edge | tired | nogoodasoth | 0,04210059 | tired--nogood |
| 40 | sample | edge | restl | lonl | 0,04206362 | restl--lonl |
| 2 | sample | edge | miser | tired | 0,04177953 | miser--tired |

| | | | | | | |
|---|---|---|---|---|---|---|
| 23 | sample | edge | enjoy | selfhate | 0,03987814 | enjoy--selfhate |
| 59 | sample | edge | restl | nogoodasoth | 0,03737985 | restl--nogooda |
| 56 | sample | edge | miser | nogoodasoth | 0,03654242 | miser--nogooc |
| 21 | sample | edge | cried | concent | 0,03628825 | cried--concent |
| 69 | sample | edge | tired | evthwrong | 0,03356827 | tired--evthwrc |
| 9 | sample | edge | tired | nogood | 0,03113123 | tired--nogood |
| 48 | sample | edge | tired | nolove | 0,03078077 | tired--nolove |
| 20 | sample | edge | nogood | concent | 0,0304577 | nogood--conce |
| 45 | sample | edge | badpers | lonl | 0,02932345 | badpers--lonl |
| 24 | sample | edge | tired | selfhate | 0,02790736 | tired--selfhate |
| 70 | sample | edge | restl | evthwrong | 0,02633294 | restl--evthwro |
| 28 | sample | edge | concent | selfhate | 0,02521691 | concent--selfh |
| 41 | sample | edge | nogood | lonl | 0,02256809 | nogood--lonl |
| 30 | sample | edge | enjoy | badpers | 0,01694351 | enjoy--badper |
| 31 | sample | edge | tired | badpers | 0,01674452 | tired--badpers |
| 34 | sample | edge | cried | badpers | 0,0165025 | cried--badpers |
| 67 | sample | edge | miser | evthwrong | 0,01572811 | miser--evthwr |
| 22 | sample | edge | miser | selfhate | 0,01232956 | miser--selfhate |
| 33 | sample | edge | nogood | badpers | 0,01094399 | nogood--badp |
| 12 | sample | edge | enjoy | cried | 0 | enjoy--cried |
| 13 | sample | edge | tired | cried | 0 | tired--cried |
| 25 | sample | edge | restl | selfhate | 0 | restl--selfhate |
| 38 | sample | edge | enjoy | lonl | 0 | enjoy--lonl |
| 46 | sample | edge | miser | nolove | 0 | miser--nolove |
| 49 | sample | edge | restl | nolove | 0 | restl--nolove |
| 52 | sample | edge | concent | nolove | 0 | concent--nolov |
| 57 | sample | edge | enjoy | nogoodasoth | 0 | enjoy--nogooc |
| 47 | sample | edge | enjoy | nolove | -0,01269081 | enjoy--nolove |
| 61 | sample | edge | cried | nogoodasoth | -0,01570354 | cried--nogood |

This table depicts the edge weights of the two nodes as regularized partial correlations:
Tired = Felt tired; selfhate = I hated myself; restl = Restless;
no love = Nobody really loved me; nogoodasoth = Not as good as other kids;
nogood = I was no good anymore; miser = Miserable/unhappy; lonl = Felt lonely;
evthwrong = Did everything wrong; enjoy = Did not enjoy anything; cried = Cried a lot;
concent = Hard to concentrate; badpers = I was a bad person

BMJ Publishing Group Limited (BMJ) disclaims all liability and responsibility arising from any reliance placed on this supplemental material which has been supplied by the author(s)
