# Supplementary material for: Depressive symptom networks in the UK general adolescent population and in those looked after by local authorities
Source: BMJ Ment Health. 2023 Sep 1;26(1):e300707. doi: 10.1136/bmjment-2023-300707 (PMC10577707; doi:10.1136/bmjment-2023-300707)
Supplement: Supplementary data [file bmjment-2023-300707supp003.pdf]

	name	type	node1	node2	value	id
55	sample	edge	lonl	nolove	0,25439076	lonl--nolove
26	sample	edge	nogood	selfhate	0,2389758	nogood--selfha
36	sample	edge	selfhate	badpers	0,22796414	selfhate--badp
78	sample	edge	nogoodasoth	evthwrong	0,22372213	nogoodasoth--
15	sample	edge	nogood	cried	0,2183755	nogood--cried
6	sample	edge	tired	restl	0,21059503	tired--restl
75	sample	edge	badpers	evthwrong	0,20500725	badpers--evthv
19	sample	edge	restl	concent	0,19398987	restl--concent
66	sample	edge	nolove	nogoodasoth	0,18470318	nolove--nogoc
3	sample	edge	enjoy	tired	0,17836975	enjoy--tired
1	sample	edge	miser	enjoy	0,16900127	miser--enjoy
18	sample	edge	tired	concent	0,15564565	tired--concent
8	sample	edge	enjoy	nogood	0,14903836	enjoy--nogooc
65	sample	edge	lonl	nogoodasoth	0,14795042	lonl--nogooda
16	sample	edge	miser	concent	0,14394387	miser--concen
37	sample	edge	miser	lonl	0,14349157	miser--lonl
2	sample	edge	miser	tired	0,13194371	miser--tired
27	sample	edge	cried	selfhate	0,12508631	cried--selfhate
53	sample	edge	selfhate	nolove	0,12287802	selfhate--nolov
74	sample	edge	selfhate	evthwrong	0,12163152	selfhate--evthv
77	sample	edge	nolove	evthwrong	0,11981484	nolove--evthw
63	sample	edge	selfhate	nogoodasoth	0,11383534	selfhate--nogc
11	sample	edge	miser	cried	0,10615234	miser--cried
44	sample	edge	selfhate	lonl	0,1028904	selfhate--lonl
5	sample	edge	enjoy	restl	0,10237575	enjoy--restl
54	sample	edge	badpers	nolove	0,10123137	badpers--nolov
50	sample	edge	nogood	nolove	0,09944749	nogood--nolov
60	sample	edge	nogood	nogoodasoth	0,09819134	nogood--nogo
10	sample	edge	restl	nogood	0,09345817	restl--nogood
21	sample	edge	cried	concent	0,09214978	cried--concent
71	sample	edge	nogood	evthwrong	0,08930837	nogood--evthv
51	sample	edge	cried	nolove	0,07927634	cried--nolove
76	sample	edge	lonl	evthwrong	0,07801089	lonl--evthwror
7	sample	edge	miser	nogood	0,07446556	miser--nogooc
4	sample	edge	miser	restl	0,07086345	miser--restl
42	sample	edge	cried	lonl	0,06915973	cried--lonl
20	sample	edge	nogood	concent	0,06895556	nogood--conce
73	sample	edge	concent	evthwrong	0,06806234	concent--evthv
30	sample	edge	enjoy	badpers	0,06315552	enjoy--badper
62	sample	edge	concent	nogoodasoth	0,06084278	concent--nogc
41	sample	edge	nogood	lonl	0,0572756	nogood--lonl
43	sample	edge	concent	lonl	0,05629049	concent--lonl
22	sample	edge	miser	selfhate	0,05111565	miser--selfhat
72	sample	edge	cried	evthwrong	0,04544739	cried--evthwrc
23	sample	edge	enjoy	selfhate	0,03866623	enjoy--selfhat
32	sample	edge	restl	badpers	0,03781368	restl--badpers
56	sample	edge	miser	nogoodasoth	0,03592932	miser--nogooc
17	sample	edge	enjoy	concent	0,02701748	enjoy--concen
39	sample	edge	tired	lonl	0,02502602	tired--lonl

68	sample	edge	enjoy	evthwrong	0,02379409	enjoy--evthwr
31	sample	edge	tired	badpers	0,02363304	tired--badpers
45	sample	edge	badpers	lonl	0,02348256	badpers--lonl
33	sample	edge	nogood	badpers	0,02137029	nogood--badp
64	sample	edge	badpers	nogoodasoth	0,02117421	badpers--nogc
47	sample	edge	enjoy	nolove	0,01963169	enjoy--nolove
40	sample	edge	restl	lonl	0,01657702	restl--lonl
35	sample	edge	concent	badpers	0,01597653	concent--badp
38	sample	edge	enjoy	lonl	0,01436651	enjoy--lonl
9	sample	edge	tired	nogood	0,01159891	tired--nogood
70	sample	edge	restl	evthwrong	0,01095476	restl--evthwro
12	sample	edge	enjoy	cried	0,00771701	enjoy--cried
58	sample	edge	tired	nogoodasoth	0,00644733	tired--nogooda
14	sample	edge	restl	cried	0,00297046	restl--cried
29	sample	edge	miser	badpers	0,00230966	miser--badper
69	sample	edge	tired	evthwrong	0,00085006	tired--evthwrc
34	sample	edge	cried	badpers	0,00041172	cried--badpers
13	sample	edge	tired	cried	0	tired--cried
24	sample	edge	tired	selfhate	0	tired--selfhate
25	sample	edge	restl	selfhate	0	restl--selfhate
28	sample	edge	concent	selfhate	0	concent--selfh
46	sample	edge	miser	nolove	0	miser--nolove
49	sample	edge	restl	nolove	0	restl--nolove
52	sample	edge	concent	nolove	0	concent--nolov
57	sample	edge	enjoy	nogoodasoth	0	enjoy--nogooc
59	sample	edge	restl	nogoodasoth	0	restl--nogooda
61	sample	edge	cried	nogoodasoth	0	cried--nogood
67	sample	edge	miser	evthwrong	0	miser--evthwr
48	sample	edge	tired	nolove	-0,02049016	tired--nolove

This table depicts the edge weights of the two nodes as regularized partial correlations:
Tired = Felt tired; selfhate = I hated myself; restl = Restless;
no love = Nobody really loved me; nogoodasoth = Not as good as other kids;
nogood = I was no good anymore; miser = Miserable/unhappy; lonl = Felt lonely;
evthwrong = Did everything wrong; enjoy = Did not enjoy anything; cried = Cried a lot;
concent = Hard to concentrate; badpers = I was a bad person
