# Supplementary material for: Depressive symptom networks in the UK general adolescent population and in those looked after by local authorities
Source: BMJ Ment Health. 2023 Sep 1;26(1):e300707. doi: 10.1136/bmjment-2023-300707 (PMC10577707; doi:10.1136/bmjment-2023-300707)
Supplement: Supplementary data [file bmjment-2023-300707supp004.pdf]

	name	type	node1	node2	value	id
36	sample	edge	selfhate	badpers	0,3020945	selfhate--badp
55	sample	edge	lonl	nolove	0,27589851	lonl--nolove
16	sample	edge	miser	concent	0,2517546	miser--concen
15	sample	edge	nogood	cried	0,24950848	nogood--cried
50	sample	edge	nogood	nolove	0,23573947	nogood--nolov
11	sample	edge	miser	cried	0,22217007	miser--cried
75	sample	edge	badpers	evthwrong	0,1916863	badpers--evth
53	sample	edge	selfhate	nolove	0,17016634	selfhate--nolo
54	sample	edge	badpers	nolove	0,16993505	badpers--nolo
6	sample	edge	tired	restl	0,16223699	tired--restl
39	sample	edge	tired	lonl	0,16215958	tired--lonl
70	sample	edge	restl	evthwrong	0,15813937	restl--evthwro
64	sample	edge	badpers	nogoodasoth	0,14967915	badpers--nogo
44	sample	edge	selfhate	lonl	0,14080058	selfhate--lonl
78	sample	edge	nogoodasoth	evthwrong	0,13348334	nogoodasoth--
3	sample	edge	enjoy	tired	0,13086422	enjoy--tired
74	sample	edge	selfhate	evthwrong	0,13062533	selfhate--evth
19	sample	edge	restl	concent	0,12995095	restl--concent
20	sample	edge	nogood	concent	0,11844578	nogood--conc
26	sample	edge	nogood	selfhate	0,11522867	nogood--selfh
42	sample	edge	cried	lonl	0,10988971	cried--lonl
62	sample	edge	concent	nogoodasoth	0,1052527	concent--nogo
66	sample	edge	nolove	nogoodasoth	0,09977449	nolove--nogoo
56	sample	edge	miser	nogoodasoth	0,09865968	miser--nogood
71	sample	edge	nogood	evthwrong	0,0926464	nogood--evthv
33	sample	edge	nogood	badpers	0,08917917	nogood--badp
72	sample	edge	cried	evthwrong	0,08366152	cried--evthwro
68	sample	edge	enjoy	evthwrong	0,08165839	enjoy--evthwr
65	sample	edge	lonl	nogoodasoth	0,08099595	lonl--nogooda
34	sample	edge	cried	badpers	0,07574174	cried--badpers
21	sample	edge	cried	concent	0,07212264	cried--concent
2	sample	edge	miser	tired	0,07150038	miser--tired
28	sample	edge	concent	selfhate	0,07010446	concent--selfh
37	sample	edge	miser	lonl	0,06888656	miser--lonl
5	sample	edge	enjoy	restl	0,06180981	enjoy--restl
63	sample	edge	selfhate	nogoodasoth	0,06106199	selfhate--nogo
45	sample	edge	badpers	lonl	0,05054121	badpers--lonl
58	sample	edge	tired	nogoodasoth	0,04566872	tired--nogooda
76	sample	edge	lonl	evthwrong	0,04506143	lonl--evthwror
1	sample	edge	miser	enjoy	0,03950719	miser--enjoy
43	sample	edge	concent	lonl	0,03899568	concent--lonl
29	sample	edge	miser	badpers	0,03643687	miser--badper
69	sample	edge	tired	evthwrong	0,03493107	tired--evthwro
13	sample	edge	tired	cried	0,02847542	tired--cried
73	sample	edge	concent	evthwrong	0,02770635	concent--evth
77	sample	edge	nolove	evthwrong	0,02627214	nolove--evthw
17	sample	edge	enjoy	concent	0,02605682	enjoy--concen
12	sample	edge	enjoy	cried	0,02430141	enjoy--cried
14	sample	edge	restl	cried	0,01913385	restl--cried

18	sample	edge	tired	concent	0,01676422	tired--concent
59	sample	edge	restl	nogoodasoth	0,01624937	restl--nogooda
4	sample	edge	miser	restl	0,01089991	miser--restl
25	sample	edge	restl	selfhate	0,00872165	restl--selfhate
51	sample	edge	cried	nolove	0,00812223	cried--nolove
10	sample	edge	restl	nogood	0,00723462	restl--nogood
61	sample	edge	cried	nogoodasoth	0,00573511	cried--nogood
7	sample	edge	miser	nogood	0	miser--nogooc
8	sample	edge	enjoy	nogood	0	enjoy--nogooc
9	sample	edge	tired	nogood	0	tired--nogood
22	sample	edge	miser	selfhate	0	miser--selfhat
24	sample	edge	tired	selfhate	0	tired--selfhate
27	sample	edge	cried	selfhate	0	cried--selfhate
32	sample	edge	restl	badpers	0	restl--badpers
35	sample	edge	concent	badpers	0	concent--badp
38	sample	edge	enjoy	lonl	0	enjoy--lonl
40	sample	edge	restl	lonl	0	restl--lonl
41	sample	edge	nogood	lonl	0	nogood--lonl
46	sample	edge	miser	nolove	0	miser--nolove
47	sample	edge	enjoy	nolove	0	enjoy--nolove
48	sample	edge	tired	nolove	0	tired--nolove
49	sample	edge	restl	nolove	0	restl--nolove
52	sample	edge	concent	nolove	0	concent--nolo
57	sample	edge	enjoy	nogoodasoth	0	enjoy--nogooc
60	sample	edge	nogood	nogoodasoth	0	nogood--nogo
67	sample	edge	miser	evthwrong	0	miser--evthwr
30	sample	edge	enjoy	badpers	-0,00987258	enjoy--badper
23	sample	edge	enjoy	selfhate	-0,02199063	enjoy--selfhat
31	sample	edge	tired	badpers	-0,02680966	tired--badpers

This table depicts the edge weights of the two nodes as regularized partial correlations:
Tired = Felt tired; selfhate = I hated myself; restl = Restless;
no love = Nobody really loved me; nogoodasoth = Not as good as other kids;
nogood = I was no good anymore; miser = Miserable/unhappy; lonl = Felt lonely;
evthwrong = Did everything wrong; enjoy = Did not enjoy anything; cried = Cried a lot;
concent = Hard to concentrate; badpers = I was a bad person
